# Meta-Analysis of Yield-Related and N-Responsive Genes Reveals Chromosomal Hotspots, Key Processes and Candidate Genes for Nitrogen-Use Efficiency in Rice

**DOI:** 10.3389/fpls.2021.627955

**Published:** 2021-06-08

**Authors:** Supriya Kumari, Narendra Sharma, Nandula Raghuram

**Affiliations:** University School of Biotechnology, Guru Gobind Singh Indraprastha University, Dwarka, India

**Keywords:** nitrogen use efficiency, N-response, yield, QTL, rice, transcription factors, photosynthesis, transpiration

## Abstract

Nitrogen-use efficiency (NUE) is a function of N-response and yield that is controlled by many genes and phenotypic parameters that are poorly characterized. This study compiled all known yield-related genes in rice and mined them from the N-responsive microarray data to find 1,064 NUE-related genes. Many of them are novel genes hitherto unreported as related to NUE, including 80 transporters, 235 transcription factors (TFs), 44 MicroRNAs (miRNAs), 91 kinases, and 8 phosphatases. They were further shortlisted to 62 NUE-candidate genes following hierarchical methods, including quantitative trait locus (QTL) co-localization, functional evaluation in the literature, and protein–protein interactions (PPIs). They were localized to chromosomes 1, 3, 5, and 9, of which chromosome 1 with 26 genes emerged as a hotspot for NUE spanning 81% of the chromosomes. Further, co-localization of the NUE genes on NUE-QTLs resolved differences in the earlier studies that relied mainly on N-responsive genes regardless of their role in yield. Functional annotations and PPIs for all the 1,064 NUE-related genes and also the shortlisted 62 candidates revealed transcription, redox, phosphorylation, transport, development, metabolism, photosynthesis, water deprivation, and hormonal and stomatal function among the prominent processes. *In silico* expression analysis confirmed differential expression of the 62 NUE-candidate genes in a tissue/stage-specific manner. Experimental validation in two contrasting genotypes revealed that high NUE rice shows better photosynthetic performance, transpiration efficiency and internal water-use efficiency in comparison to low NUE rice. Feature Selection Analysis independently identified one-third of the common genes at every stage of hierarchical shortlisting, offering 6 priority targets to validate for improving the crop NUE.

## Introduction

The Phenomenal growth in the use of fertilizers for crop production, coupled with poor nitrogen-use efficiency (NUE) is increasingly polluting the soil, water and air, which adversely affects health, biodiversity, and climate change (Sutton et al., 2019). A recent simulation study showed that a 20% increase in the crop NUE can save $743 million per year in the USA alone (Langholtz et al., 2021). Therefore, improving the crop NUE remains a highly desirable economic and environmental goal. NUE can be defined in terms of uptake/utilization or remobilization efficiencies, but it is agronomically best expressed as yield per unit nitrogen input (Raghuram and Sharma, 2019). An inability to biologically distinguish between the N-response and NUE and the poor characterization of the phenotype and genotype for NUE have hampered crop improvement (Mandal et al., 2018), till they were discovered recently (Sharma et al., 2018, 2021).

Rice has the lowest NUE among cereals (Norton et al., 2015) and therefore consumes most N-fertilizer among them. It is also the third most produced and consumed crop in the world. Further, its rich germplasm diversity and post-genomic status makes it an ideal candidate to improve the crop NUE on a global scale. The tremendous growth of rice genomics, such as the sequencing of 3,000 rice genomes (Li et al., 2014) and of rice functional genomics (Li et al., 2018), has enabled the integration of information on the available genes, germplasm and phenotypic information. Rice also has the most N-responsive transcriptomic data sets reported in any crop (Pathak et al., 2020 and the references cited therein).

Some of the important functional classes of genes involved or implicated in NUE include transporters, kinases, and transcription factors (TFs) (Vidal et al., 2020; Yang et al., 2020; Zhang et al., 2020b). Recently, Kumari and Raghuram (2020) compiled a comprehensive list of phosphatases involved in the N-response and/or NUE in crops. MicroRNAs (miRNAs) also regulate the use of nitrogen in crop plants and participate in the adaptation of crops to nitrogen deficiency (Zuluaga and Sonnante, 2019). Some of these functional classes of genes are also known to be involved in yield; over a 1,000 yield-related genes have been reported in rice so far (Li et al., 2019; Nutan et al., 2020). However, a genetic characterization of the interface between N-response and yield is lacking, especially considering that NUE is a derivative of two biological functions, N-response and yield, and many genes involved in them are well-known.

A number of studies exist on the quantitative trait loci (QTLs) associated with NUE in rice (Anis et al., 2019; Jewel et al., 2019; Mahender et al., 2019; Zhang et al., 2020b; and the references cited therein). These include both major and minor QTLs, which are categorized based on the magnitude of their effect on the phenotype under different N regimes. These QTLs for NUE can be used to co-localize the genes for further characterization (Sinha et al., 2018; Waqas et al., 2018). However, there has been an inadequate convergence of the QTL and functional genomics or reverse genetic approaches to understand the molecular basis of NUE or its associated phenotype in any crop. Previous attempts to identify NUE-candidate genes were based on co-localizing N-responsive genes on NUE-QTLs (Sinha et al., 2018) or on yield QTLs (Chandran et al., 2016), regardless of their role in yield or NUE. Others identified all the genes in the NUE-QTL region, regardless of their role in N-response, yield, or both (Jewel et al., 2019; Mahender et al., 2019).

Therefore, we searched for the genes involved in both N-response and yield as the main biological components of NUE, co-localized them on major NUE-QTLs and identified their chromosomal hotspots to narrow down the genetic basis for NUE. We also shortlisted candidate genes, associated phenotypes and biological processes for NUE and validated some of them in two contrasting rice genotypes differing in NUE.

## Materials and Methods

### Plant Materials and Growth Conditions

Two rice genotypes (*Oryza sativa* subsp. Indica) contrasting for NUE were selected based on a previous study (Sharma et al., 2018): Nidhi for low NUE and Panvel1 for high NUE. The seeds of Panvel1 were procured from Kharland rice research station, Panvel, Maharashtra, India, while the seeds of Nidhi were procured from the Indian Institute of Rice Research, Hyderabad, India. Seeds of each genotype were weighed individually and only seeds of modal weight (±0.5 mg) were used as described in previous studies (Sharma et al., 2018, 2021). They were surface sterilized with 0.1% mercuric chloride for 50 s, washed 8–10 times with ultrapure deionized water and soaked in it for 2 h before sowing in pots containing garden soil. Before sowing, the pots were saturated with Arnon–Hoagland medium (Hoagland and Arnon, 1950) containing nitrate as the sole source of N at 15 mM (normal N) or 1.5 mM (low N) level. This was achieved by using 5 mM each of KNO_3_ and Ca(NO_3_)_2_ for normal N and 0.5 mM each for low N. The media components were obtained from SRL, Mumbai, Maharashtra, India. There were five replicates/pots, each containing a single plant for each N dose. The pots were replenished with media to saturation every few days as needed. Plants were grown in the greenhouse at 28°C, 75% humidity, 40 Klux light intensity, and 12/12 h photoperiod for 21 days. The leaves of these 21-day-old plants were used to measure the physiological parameters on sunny days in five biological replicates by using a portable Licor 6400XT (LI-COR, Lincoln, NE, USA) as per the instructions of the manufacturer. The net photosynthetic rate was measured in terms of assimilated CO_2_, as μ mol CO_2_/m^2^s^1^, transpiration was measured in terms of mol (H_2_O)/m^2^s^1^, stomatal conductance was measured in terms of mol [(H_2_O) m^−2^ sec^−1^], internal water-use efficiency was measured in terms of μ mol CO_2_/mol(H_2_O), and transpiration efficiency was measured in terms of μ mol CO_2_/m mol H_2_O/m^2^/s. The Student's *t*-test was performed on the recorded physiological data.

### Data Mining for N-Responsive and Yield-Related Genes

N-responsive genes in rice were retrieved from 16 whole transcriptome microarray data sets (Supplementary Table S1). Yield-related genes in rice were compiled from 219 publications in the literature, as well as five online databases, namely, funRice Genes (https://funricegenes.github.io/), Oryzabase (https://shigen.nig.ac.jp/rice/oryzabase/), RAPDB (https://rapdb.dna. affrc.go.jp/tools/dump), Ricyer DB (http://server.malab.cn/Ricyer/index.html), and OGRO DB (http://qtaro.abr.affrc.go.jp/ogro/). The guidelines of Preferred Reporting Items for Systematic Reviews and Meta-Analyses (PRISMA, Moher et al., 2009) were followed. Only sources that were true for at least one of the following three criteria were considered: (1) Do they belong to N-responsive category? (2) Do they belong to the yield category? and (3) Are they involved in NUE? Keywords like grain, tiller angle, culm, leaf angle, dwarf, grain shape, grain size, grain weight, grains per panicle, growth period, leaf, length-to-width ratio, number of panicles per plant, panicle, panicle length, panicle number, plant height, productivity, grain filling, seed setting rate, spikelet numbers, tiller, flowering, tiller angle, biomass, and photosynthetic efficiency were considered based on the studies of Nutan et al. (2020) and Li et al. (2019). They were used to search the literature and database for yield-related genes. The differentially expressed genes (DEGs) were identified by using uniform criteria of Log_2_FC ≥ 1 and a value of *p* ≤ 0.05 with default redundancy removal criteria.

### Meta-Analysis for N-Responsive and Yield-Related Genes

All yield-related genes retrieved from the literature and databases were combined into a single yield-related data set and all N-responsive DEGs retrieved from 16 transcriptome microarrays were also combined into a separate N-responsive data set. Duplicates from both the data sets were removed and only non-redundant genes were used for Venn selection (https://bioinfogp.cnb.csic.es/tools/venny/index2.0.2.html) to identify the genes that were both N-responsive and yield-related. They were termed as NUE-related genes and used for all the downstream analyses.

### Functional Annotation and MapMan Analysis of NUE-Related Genes

Gene ontology (GO) enrichment analyses for functional annotation of NUE-related genes were performed by Expath 2.0 tool (Chien et al., 2015) using default parameters. Only statistically significant GO terms with the value of *p* < 0.05 were considered for further analyses and visualized by REVIGO (Supek et al., 2011). The MapMan 3.6.0 RC1 tool was used for analyzing the DEGs in different biological pathways (Thimm et al., 2004).

### Retrieval of Functional Classes and Identification of Target Genes for miRNA

Nitrogen-use efficiency-related genes encoding TFs were retrieved from the following databases: PlantPAN2 (http://plantpan2.itps.ncku.edu.tw/TF_list_search.php#results), RAP-DB, STIFDB (http://caps.ncbs.res.in/stifdb/), PlantTFDB (http://plan ttfdb.cbi.pku.edu.cn/index.php?sp=Osj), and RiceFrend (http://ricefrend.dna.affrc.go.jp/multi-guide-gene.html). For the prediction of TF binding sites (TFBSs), 2 kB promoter regions upstream of the translational start site of the TFs were downloaded from RAPDB and were subjected to Regulatory Sequence Analysis Tools (RSAT) (http://plants.rsat.eu). To find out the motif sequences and their enrichment in these TFs, only ≥ 8mer sequences with a significance level <0.01 were obtained from TFBSs. Tomtom v 5.1.1 tool (http://meme-suite.org/tools/tomtom; Gupta et al., 2007) was used with default settings to filter redundant motifs and define known conserved regulatory elements (CREs) based on the motif database (Arabidopsis DAP motifs). To identify possible biological and molecular functions of motifs, the GoMo tool (http://meme-suite.org/ tools/gomo) was used (Buske et al., 2010). NUE-related gene-encoding transporters were retrieved from the Rice transporters database (https://ricephylogenomics.ucdavis.edu/transporter/), RAPDB, and Transport DB2.0 (http://www.membranetransport.org/transportDB2/index.html). Similarly, NUE-related gene-encoding kinases and phosphatases were retrieved from RAPDB and i-TAK DB (http://itak.feilab.net/cgi-bin/itak/index.cgi). Plant miRNA database (PMRD, http://bioinformatics.cau.edu.cn/PMRD/) was used for searching the miRNAs that target NUE-related genes.

### Subcellular Localization of NUE-Related Gene Products

The subcellular localization of NUE-related gene-encoded proteins was predicted by using crop Proteins with Annotated Locations database (cropPAL2; https://croppal2.plantenergy.edu.au/; Hooper et al., 2016). This database contains proteins whose subcellular location is most commonly determined by fluorescent protein tagging of live cells or mass spectrometry detection from the metadata for protein and the published literature studies (Hooper et al., 2016).

### Protein–Protein Interaction Network Construction

Construction of protein–protein interaction **(**PPI) networks was undertaken by using different subsets of genes to find interacting proteins in the context of (a) NUE-related genes and (b) shortlisted candidate genes for NUE. For this purpose, the interacting proteins for the NUE-related genes and shortlisted candidate genes for NUE were retrieved from the STRING (https://string-db.org) database. Interactions demonstrated by experimental and combined scores were considered to map the query genes onto the PPI networks. Based on the experimental score and the combined score, PPI networks for NUE-related genes and shortlisted candidate genes for NUE were constructed by using Cytoscape version 3.8 (Shannon et al., 2003), and the expression values of DEGs were used to color code the nodes. Molecular complexes were detected by using the MCODE plug in Cytosacpe.

### Co-localization of NUE-Related Genes Onto NUE-QTLs

Nitrogen-use efficiency QTLs reported under different nitrogen regimes were compiled from the literature, and GRAMENE DB (https://archive.gramene.org/db/markers/marker_view) and were used to retrieve the positions of markers flanking these QTLs. Only major QTLs having phenotypic variance >10% were considered, and those identified as relative QTLs were considered under different doses of N (Sinha et al., 2018). N-responsive and yield-related DEGs identified in the present study were co-localized to these NUE-QTL regions.

### *In silico* Expression Analysis of Shortlisted Candidate Genes for NUE

For tissue/organ-specific expression of N-responsive yield-related DEGs at different stages of the rice life cycle, the Rice Expression Profile Database (http://ricexpro.dna.affrc.go.jp/GGEP/gene-search.php) was used.

### Feature Selection Analysis

The recursive feature elimination method of the Feature Selection Analysis was carried out by using the Python Scipy library to bioinformatically rank the genes based on their N-response and related to yield. The commonly identified genes based on the N-response and related to yield in rice were subjected to the Feature Selection Analysis with their Log_2_FC and values of *p* to determine the genes/features that contribute to NUE.

## Results

**Figure 1:**
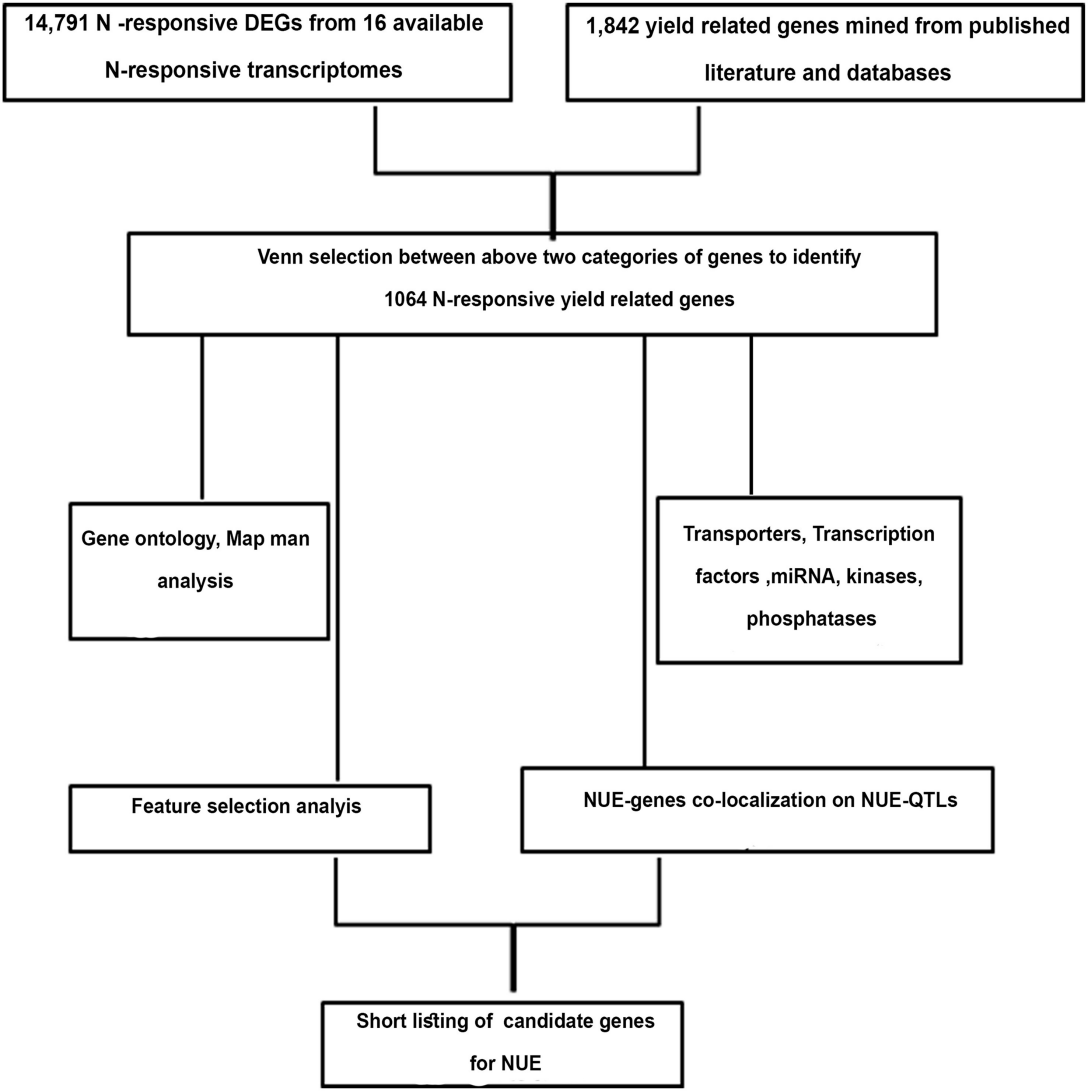
Flowchart used for identification and shortlisting of candidate genes for nitrogen-use efficiency (NUE) in rice.

**Figure 2:**
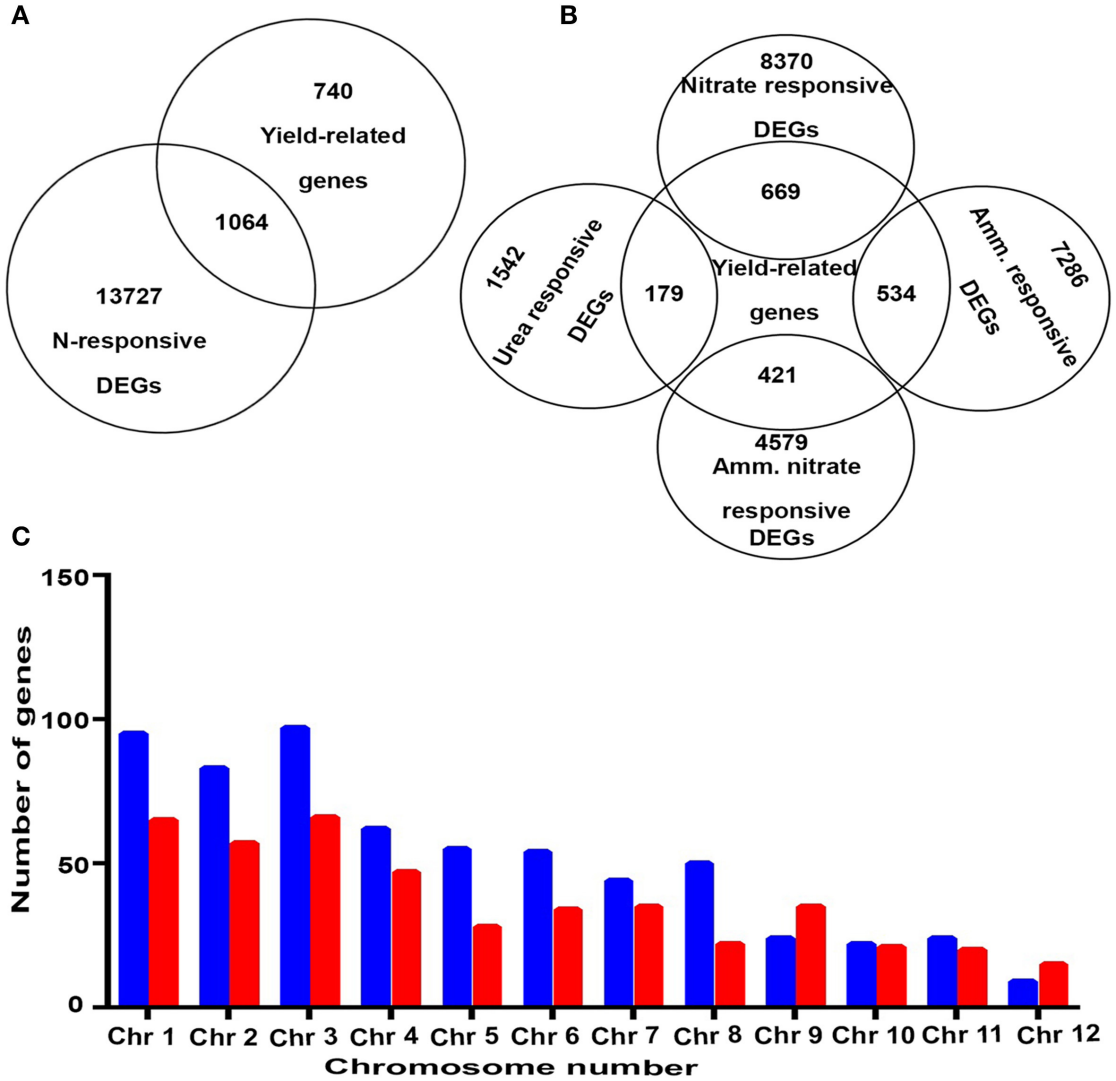
**(A)** A Venn diagram showing exclusively yield-related genes, N-responsive and yield-related genes, and exclusively N-responsive genes. **(B)** A Venn diagram showing common genes between yield and those that respond to specific N-forms. **(C)** A bar graph depicting chromosomal localization and upregulated and downregulated NUE-related genes. Blue color represents upregulated genes and red color represents downregulated genes.

Nitrogen-use efficiency is not a biological measure by itself, but a derivative of biological measures such as the N-response and yield. Many genes involved in the yield or N-response are known separately, but a comprehensive listing and analysis of genes that are both N-responsive and yield-related were not available. Therefore, we identified 1,064 common genes between 14,791 N-responsive genes and 1,842 yield-related genes known in rice and analyzed them as outlined in Figures 1, 2A. These 1,064 genes were termed as NUE-related genes (Supplementary Table S2) and were used for further downstream analyses. As these genes were derived from transcriptomic analyses using different N-forms, separate Venn selections revealed the breakup of yield-related genes for each N-form. As shown in Figure 2B, the highest numbers of yield-related genes were found for nitrate, followed by ammonium, ammonium nitrate and urea. Nevertheless, all 1,064 genes are of interest for further molecular characterization of NUE, especially considering that field soils tend to contain dynamic mixtures of multiple N-forms. Their chromosomal localization revealed that most of these were present on chromosome 3, followed by chromosomes 1, 2, and 4, while chromosome 12 harbors the least number of genes. Further, chromosomes 1–8, 10, and 11 contain more N-upregulated genes in comparison to N-downregulated genes, while chromosomes 9 and 12 have more N-downregulated genes in comparison to N-upregulated genes (Figure 2C).

### Biological Pathways and Subcellular Locations of NUE-Related Genes

**Figure 3:**
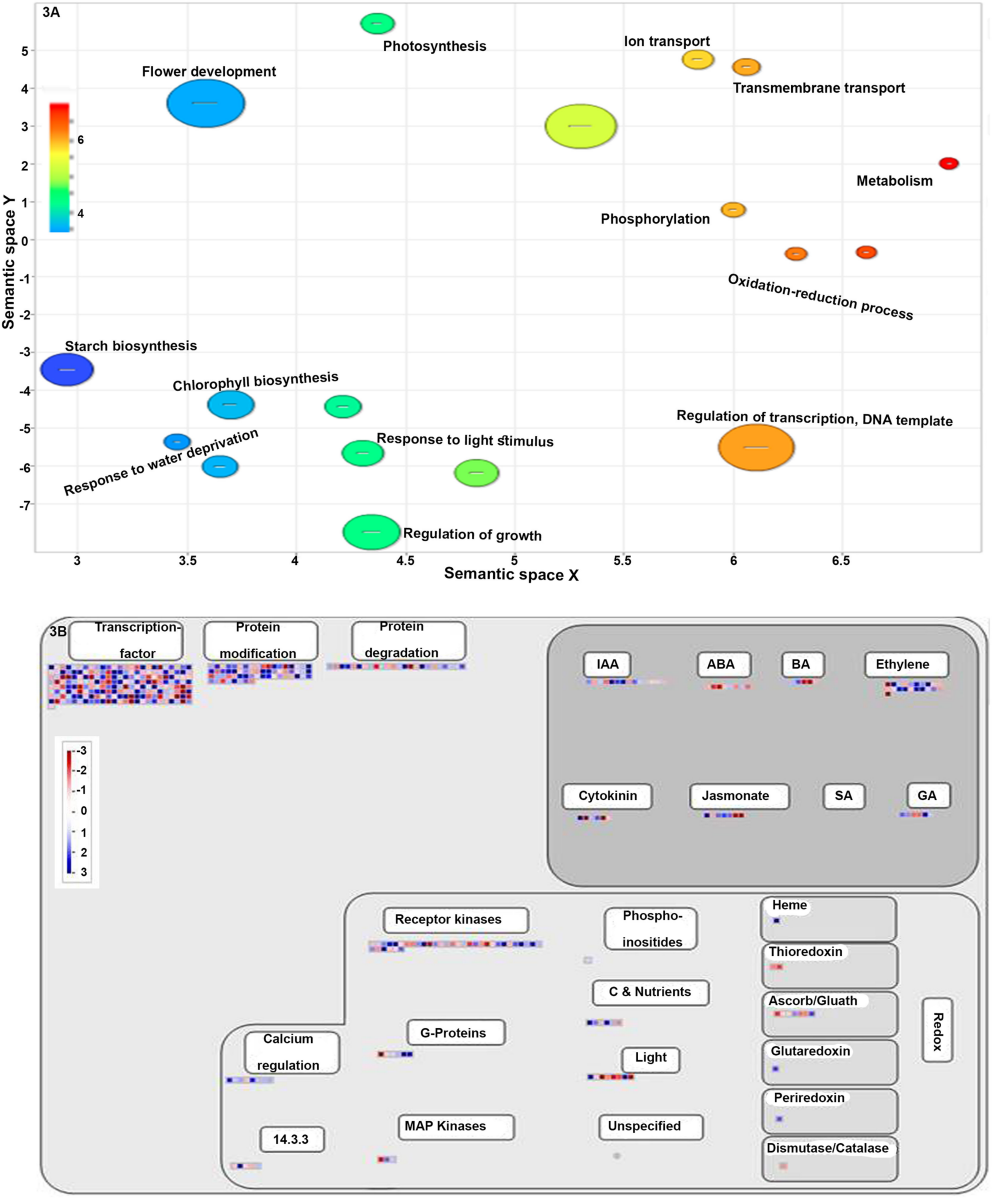
**(A)** A scatter plot showing the enriched gene ontology (GO) terms in the biological process. Different shades in circles indicate the difference in the value of *P* as indicated in scale. All the terms are significantly over-represented at *p* < 0.01. **(B)** MapMan-based classification of NUE-related genes involved in regulation. Red and blue color boxes represent the upregulated and downregulated NUE-genes, respectively.

Gene ontology enrichment analysis for the biological process showed that most of the NUE-related genes were involved in the regulation of transcription (16.17%), followed by oxidation-reduction (12.6%), phosphorylation (7.62%), flower development (3.56%) and photosynthesis (2.13%). Among the rest, 0.2% of the NUE-related genes were involved in each of the 73 different processes, including seed maturation, water homeostasis, asparagine biosynthetic process, seed germination, stomatal closure, auxin-mediated signaling pathway, photoperiodism and root and shoot development. The details of GO-enrichment analyses are provided in Supplementary Table S3. The top 25 statistically significant biological processes (*p* < 0.05) in terms of the number of NUE-related genes were visualized by using REVIGO (http://revigo.irb.hr/) (Figure 3A).

Most of the above process annotations, which include transcriptional regulation, transport, protein modification and hormone metabolism (Supplementary Table S4 and Figure 3B), were validated when all the identified NUE-related genes and their expression values were mapped into MapMan. Many NUE-related genes were also mapped to sucrose metabolism, nitrate metabolism and photorespiration, among others, suggesting a crosstalk between these pathways (Supplementary Table S5 and Supplementary Figure 1A). MapMan analysis also revealed many other DEGs involved in the development as well as in abiotic stress and biotic stress responses (Supplementary Table S6 and Supplementary Figure 1B). Subcellular localization of the proteins encoded by all the 1,064 NUE-related genes revealed that most of them are localized in four major compartments led by the nucleus, followed by cytosol, plasma membrane and plastid. The least of them are located in the peroxisome and vacuole (Supplementary Table S7 and Supplementary Figure 1C).

### NUE-Related TFs and Their Binding Motifs

To understand the role of transcriptional regulation in NUE, we identified 60 classes of TFs encoded by 237 NUE-related genes. The details of their genes, families, functions, and references are provided in Supplementary Table S8. They include 15 major classes (≥5 genes), totaling 166 genes and 45 minor classes (≤4 genes) totaling 71 genes.

**Figure 4:**
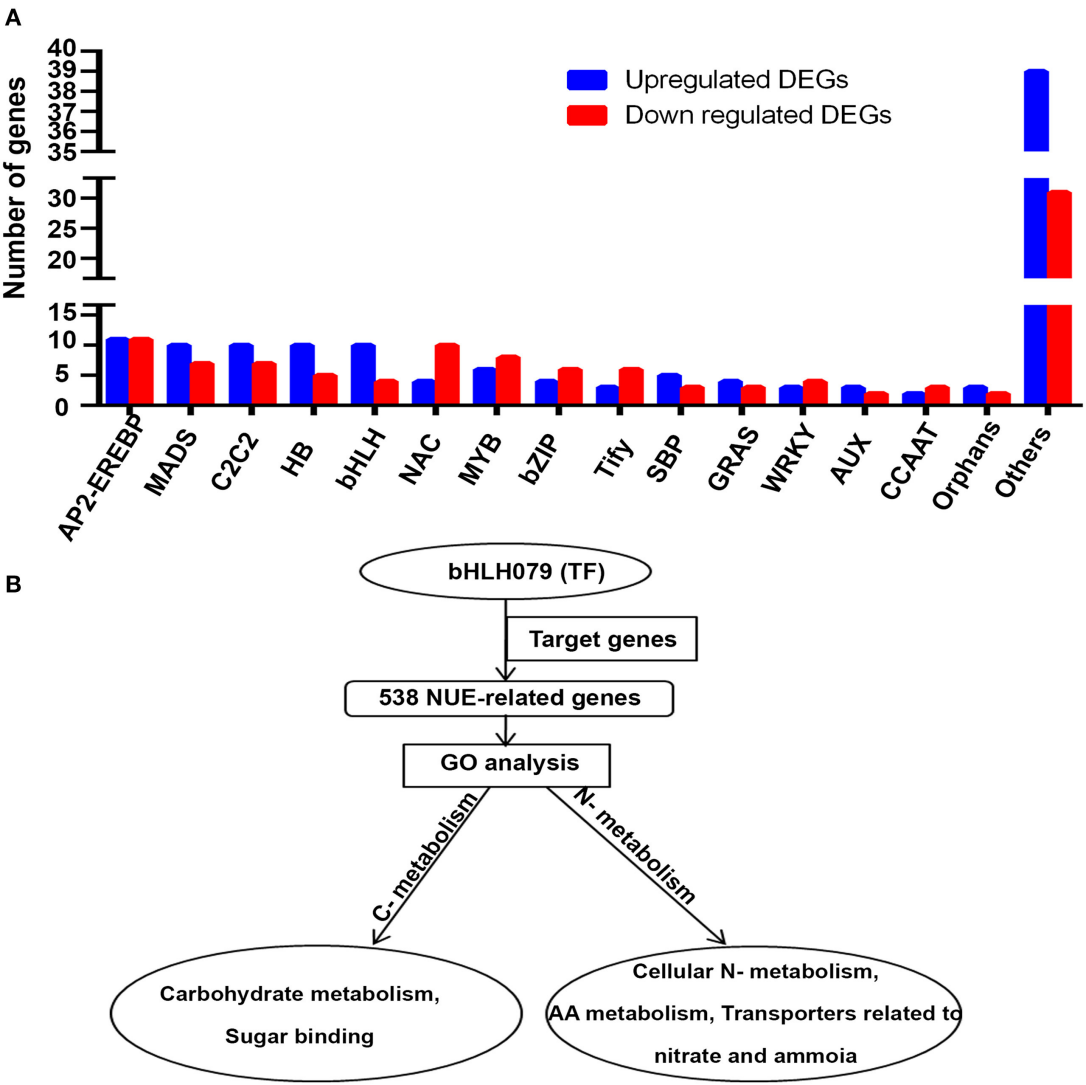
**(A)** Upregulated and downregulated NUE-related transcription factors (TFs). **(B)** Role of TF OsbHLH079 and its target genes in regulating carbon–nitrogen metabolism. AP2-EREBP, a ptela-2/ethylene-responsive element binding protein; MADS, Minichromosome maintenance1, agamous deficiens and serum response factor; C2C2, CO-like, Con stans-like zinc finger family; HB, Homeobox TF family; bHLH, Basic helix-loop-helix family; NAC, NAM, ATAF, and CUC; MYB, myeloblastosis; bZIP, Basic leucine zipper; SBP, Squamosa promoter binding protein-like; GRAS, Gibberellic-acid insensitive (GAI), repressor of GAI (RGA), and scarecrow (SCR); WRKY, AUX, Auxin/indole-3-acetic acid; CCAT, calcium channel associated transcriptional regulator.

Among these, minichromosome maintenance1, agamous deficiens and serum response factor (MADS), CO-like, con stans-like zinc finger family (C2C2), homeobox TF family (HB), basic helix-loop-helix family (bHLH), squamosa promoter binding protein-like (SBP) proteins, gibberellic-acid insensitive (GAI), repressor of GAI (RGA) and scarecrow (SCR) (GRAS), auxin/indole-3-acetic acid (AUX), and orphans were among the abundant N-upregulated TF families, while NAM, ATAF, and CUC (NAC), myeloblastosis (MYB), basic leucine zipper (Bzip), Tify, WRKY, and CCAAT TF families had more N-downregulated genes than N-upregulated genes. Only a ptela-2/ethylene-responsive element binding protein (AP2-EREBP) had an equal number of N-upregulated and N-downregulated genes. N-upregulated TF families dominated in the minor TF category, which were clubbed together and shown as “other” in Figure 4A. This suggests that a large repertoire of TFs of several major and minor families are involved in the N-response as well as are yield-related and are therefore of interest for NUE. As only two of them, i.e., *Dof1* and *OsGRF4*, are known to be associated with NUE, this analysis offers many more TFs as candidates for further validation and shortlisting to improve NUE in rice and possibly other crops.

Eight of the identified TFs are completely novel and not yet functionally validated. They are *ASD1, OsbHLH118, OsbHLH090, BZR1, OsERF27, OsLF, OsERF65*, and *OsbHLH186*. Among others, 193 TFs are hitherto unknown to be related to the NUE or N-response or yield, though they were associated with other functions. Thirty additional TFs are known only for yield and three others are known to be N-responsive.

Our analysis of the promoter regions (up to 2 kB upstream) of all 1,064 NUE-related genes for binding motifs of TFs identified above revealed a total of 50 enriched motifs (Supplementary Table S9). Majority of these motifs have binding sites for AP2-EREBP and Teosinte branched1/Cincinnata/proliferating cell factor (TCP) TFs, followed by Cys_2_His_2_ (C2H2), NIN-LIKE PROTEIN (NLP), MYB, NAC, SBP, and bHLH TFs, indicating that the NUE-related TFs are themselves regulated by these families of TFs. Annotation analysis of these motifs revealed many interesting biological and molecular functions, which include GCTAGCTA (NAC TF) for auxin stimulus and response to abscisic acid stimulus, GAGCTAGC (C2C2-GATA) for wounding response, CCGCGGCG (AP2-EREBP) for mitochondrial transport, GCGCGCGC (BZR) for stomatal complex morphogenesis, among others, but a few were not annotated. Moreover, these motifs were also involved in functions such as lipid binding, structural constituent of the ribosome, microtubule motor activity and ATPase activity. All of these CREs are novel NUE-related CREs in rice (Supplementary Table S10).

### Transcription Factor *OsbHLH079* Could Be Important in N Source-Sink Metabolism

One of the NUE-related TFs identified in this study, *OsbHLH079*, is not well-characterized in terms of its target genes and its role in regulating NUE in rice. However, its orthologs in Arabidopsis have been reported to target 863 genes (Brooks et al., 2019). We used Ensembl Plants (http://plants.ensembl.org) to retrieve their orthologs in rice. Interestingly, 538 of them were among the 1,064 NUE-related rice genes identified in this study. In other words, over half of all the NUE-related genes identified in rice are targets of this TF, *OsbHLH079*. AgriGO GO analysis of these 538 target genes revealed their involvement mainly in cellular nitrogen metabolism, amino acid metabolism, carbohydrate metabolism, and transporters related to nitrate/ammonia and sugar binding. This indicates their primary role in carbon and nitrogen metabolism, as shown in Figure 4B. Their detailed functional annotations have been provided in Supplementary Table S11.

### NUE-Related Transporters

**Figure 5:**
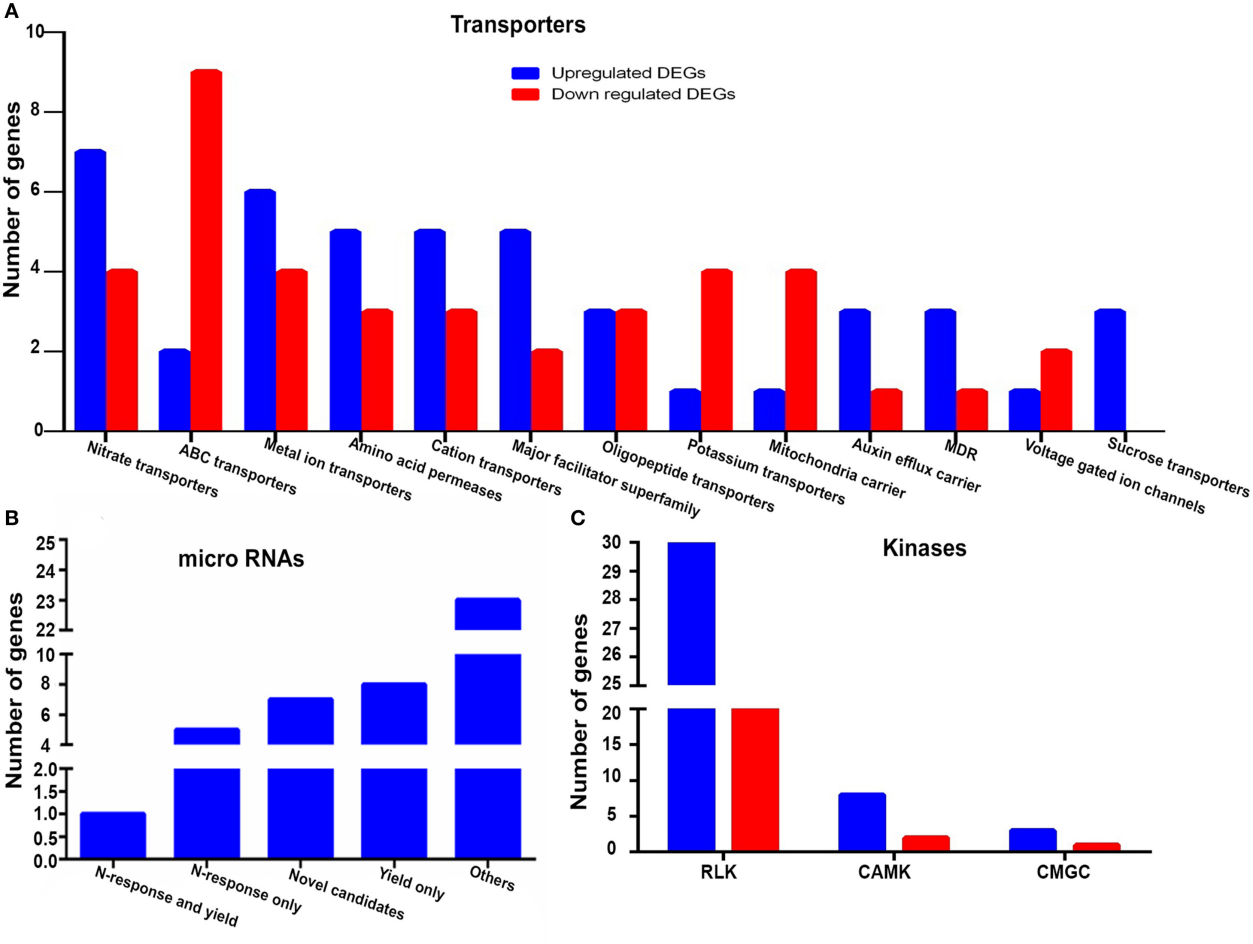
**(A)** Family-wise classification and distribution and upregulated and downregulated NUE-related transporters **(B)** microRNAs (miRNA) belonging to N-response, yield, N-response and yield, other categories and reporting for the first time (novel NUE-genes) **(C)** Upregulated and downregulated major families of NUE-related kinases.

We identified 85 gene-encoding transporters as NUE-related, since they were both N-responsive and yield-related. These belong to 13 major families (Figure 5A). The details of their genes, families, functions, and references are provided in Supplementary Table S12. Among these, the families that have more N-upregulated genes than N-downregulated genes include nitrate transporters, a metal ion, amino acid permease, cation transporters, a major facilitator superfamily, an auxin efflux carrier, and multidrug resistant (MDR). Conversely, the families that have more N-downregulated genes than N-upregulated genes include ABC and K^+^ transporters, a mitochondrial carrier, and a voltage-gated ion channel. Oligopeptide transporters have an equal number of N-upregulated and N-downregulated genes, while sucrose transporters have only N-upregulated genes (Figure 5A). This suggests that different types of transporters are involved in N-response as well as yield and are therefore of interest for NUE. While five of them are known to be associated with NUE, our analysis identified 80 more transporters as novel candidates to be validated for their potential to improve NUE. Six of them are completely novel and functionally unvalidated. These are *OsSTA85, OsEIN2.2, OsABCB15, OsABCG16, OsEIN2.2*, and *OsSTA234*. Other 61 are hitherto unknown to be related to N-response or yield or NUE though they were associated with other functions. Three others were reported for N-response only (*OsNRT2.4, OsProT1*, and *OsNPF2.4*), and seven were known for yield only, but not for both (*PIN5B, OSAAP5, OsSUT1, OsPUP4, OsAAP10D, NPF7.1* and *OsABCG18*). Thus, 80 of the 85 transporters found are novel candidates for NUE to be used for further validation.

### NUE-Related miRNAs Mainly Target Genes for Amino Acid Metabolism

An interesting subset of the 1,064 NUE-related rice genes identified in this study comprised of 44 unique miRNAs that target 69 NUE-related genes, of which 35 were N-upregulated and 34 were N-downregulated. The details of their genes and functions along with references are provided in Supplementary Table S13. The pathway analysis by ExPath2.0 (http://expath.itps.ncku.edu.tw/) revealed that, out of six significant pathways, three pathways were related to amino acid metabolism involving arginine, proline, beta-alanine, and tryptophan, which indicate the role of these miRNAs in regulating N-metabolism. Other interesting pathways regulated by these small regulatory molecules were related to diterpenoid biosynthesis, limonene and pinene degradation and plant hormone signal transduction. Out of all 44 miRNAs, 7 miRNAs were completely novel and unannotated: osa-miR170a, osa-miR1847.2, osa-miR2095-3p, osa-miR2101-5p, osa-miR2102-3p, osa-miR2104, and osa-miR444a.1. Twenty-three other miRNAs were found to be involved in functions other than N-response/yield (Supplementary Table S13). Five other miRNAs have been reported for N-response but not in yield: osa-miR156ab, osa-miR156k, osa-miR528, osa-miR529b, and osa-miR399i. Nine other miRNAs were known for yield but not for N-response: osa-miR1317, osa-miR1424, osa-miR1427, osa-miR1436, osa-miR1439, osa-miR1440, osa-miR1863, osa-miR818a, and osa-miR399 (Figure 5B and Supplementary Table S13). Two miRNAs were found to be both N-responsive and yield-related: osa-miR1318 and osa-miR156a. All other categories of miRNAs emerged as novel NUE-related genes for further validation.

### NUE-Related Kinases and Phosphatases

Protein kinases and phosphatases are known to play an important role in N-response and NUE in crops (Fataftah et al., 2018; Hsieh et al., 2018; Jiang et al., 2018; Xiong et al., 2019; Kumari and Raghuram, 2020). In this study, we identified 91 NUE-related gene-encoding kinases and 9 other gene-encoding phosphatases. The identified kinases were classified according to i-TAQ database and the database by Vij et al. (2008). These include major classes, such as 54 receptor-like kinases (RLKs), 10 CAMK:Ca2+/calmodulin-dependent protein kinases (CAMKs), 4 cyclin-dependent kinases (CDKs), mitogen-activated protein kinases (MAPKs), glycogen synthase kinase (GSK), and CDC-like kinase (CLK) (CMGC). Besides this, there were 23 other kinases belonging to minor classes (Supplementary Table S14). The details of their genes, families, functions, and references are provided in Supplementary Table S14. All major classes of NUE-related kinases showed a greater number of N-upregulated genes (Figure 5C). Out of these 91 kinases, 22 RLKs were annotated as specific kinases but not functionally validated at all, while 53 are known to be related to functions other than N-response or yield or both but hitherto unknown to be potentially NUE-related (Supplementary Table S14). Among the rest, 16 kinases are reported for yield-related traits, but not for N-response or NUE.

Nine phosphatases identified among NUE-related genes are Fructose-1,6-bisphosphatase, Type II inositol-1,4,5-trisphosphate 5-phosphatase 12, Type I inositol-1,4,5-trisphosphate 5-phosphatase CVP2, Chloroplast inorganic pyrophosphatase, Vacuolar H+-translocating pyrophosphatase, Fructose-1,6-bisphosphatase class 1/Sedoheputulose-1,7-bisphosphatase, Pyrophosphate-energized vacuolar membrane proton pump, Inositol phosphatase-like protein, and *PP2C68* (Supplementary Table 15). Among these, six genes showed N-upregulation while the three genes showed N-downregulation. The details of their genes, families, functions, and references are provided in Supplementary Table S15. Out of these nine phosphatases, eight are potential candidates to be validated for their role in NUE, including *PP2C68*, which has been reported separately for the N-response (Hsieh et al., 2018) and yield (Li et al., 2013) but not specifically implicated in NUE. Among others, Type I inositol-1,4,5-trisphosphate 5-phosphatase *CVP2* is a novel candidate yet to be functionally validated. Four others, *OsPPa6, OscFBP2, OVP1*, and *NYC4*, have been reported for functions other than N-response or yield or both (Supplementary Table S15) and are therefore hitherto unknown as candidates for NUE. Two other phosphatases (similar to Type II inositol-1,4,5-trisphosphate 5-phosphatase 12 and Vacuolar H^+^ translocating pyrophosphatase, the regulation of grain chalkiness) have been reported for yield but not for N-response or NUE. Overall, this study revealed 91 kinases and 8 phosphatases of different families related to NUE, including 8 phosphatases hitherto unknown in NUE as candidates for further validation.

### Interactions of NUE-Related Proteins in C/N Metabolism and Signaling

To understand the role of protein level interactions in regulating NUE, we developed PPI networks for the 1,064 NUE-related genes identified in this study using Cytoscape 3.8. The network consisted of 525 nodes and 1,134 edges and sub-clustering using MCODE plugin-identified 18 sub-clusters (Supplementary Table S16 and Supplementary Figures 2A–R). Among these, cluster 1 has the maximum MCODE score of 7.33, while clusters 15, 16, 17, and 18 have the minimum MCODE score of 2.67. Maximum nodes (10) and edges (33) were found for cluster 1, while minimum nodes (3) and edges (3) were found for clusters 12, 13, and 14. EXPath 2.0 analysis revealed the pathways associated with these networks/clusters. They include starch and sucrose metabolism, carbon fixation in photosynthetic organisms, metabolic pathways, photosynthesis, pentose and glucuronate interconversions, pyruvate metabolism, biosynthesis of amino acids, ribosome, aminoacyl-tRNA synthesis, ubiquitin-mediated proteolysis, and plant hormone signal transduction. Their cluster-wise pathways have been provided in Supplementary Table S16. Interestingly, two of the interacting proteins encoded by *NPH1* and *LAC13/14* are not a part of the 1,064 NUE-related genes. *NPH1* was N-responsive but not known to be yield-related, while *LAC13/14* was neither involved in N-response nor in yield. Their functional annotation indicated their role in photosynthesis, flowering and stress.

### 327 NUE-Related Genes Co-localized on Major NUE-QTLs

**Figure 6:**
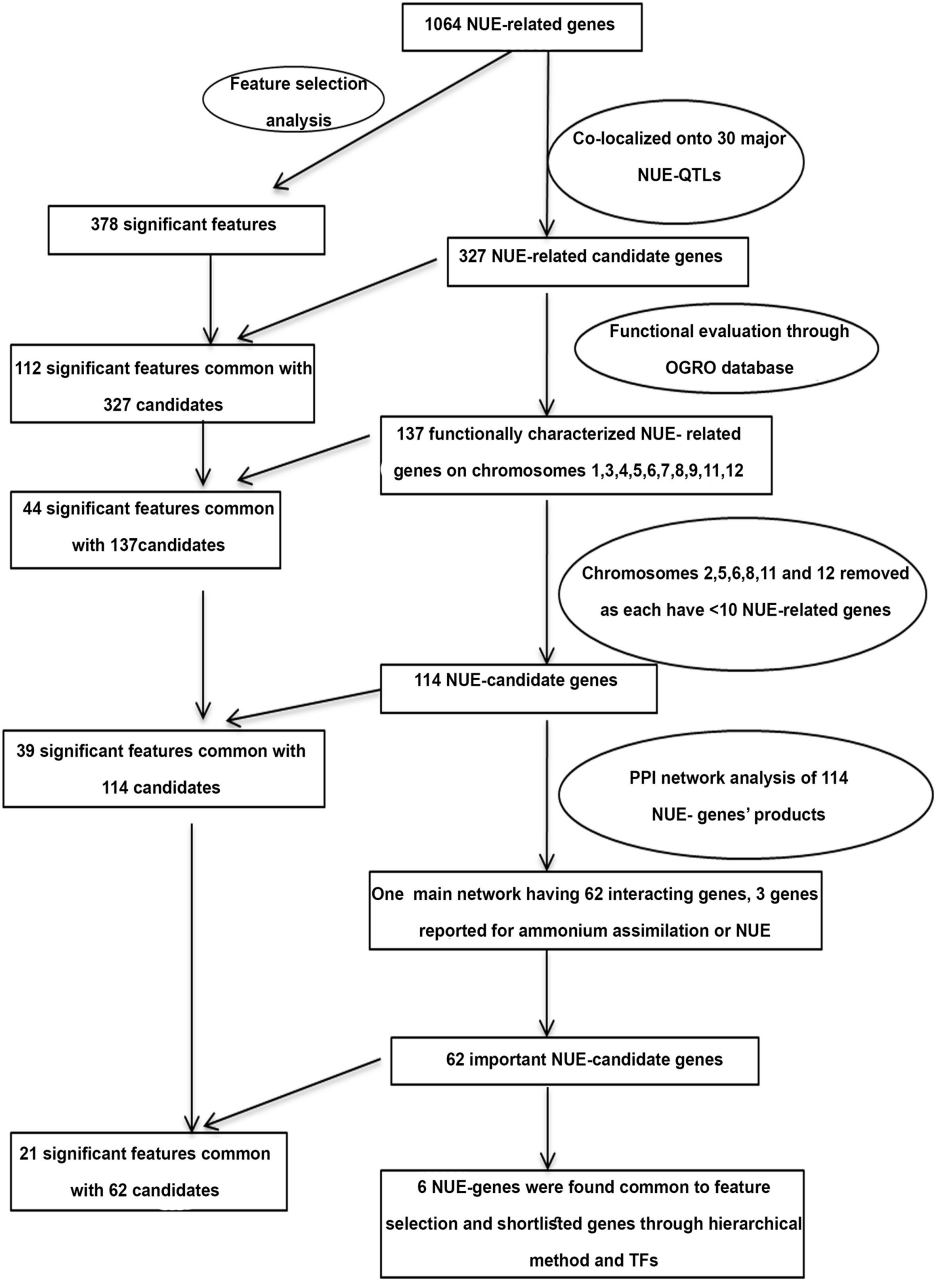
Hierarchically shorted candidate genes and independent Feature Selection method to identify NUE-candidate genes.

The hierarchical shortlisting of 1,064 NUE-related genes began with the co-localization of NUE-genes onto major NUE-QTLs as summarized in Figure 6. Using all the criteria mentioned in Section “Materials and Methods”, we compiled 30 major NUE-QTLs reported for 18 NUE-related traits, including the number of productive tillers (PTN), grain yield (GY), grain yield response (GR), harvest index (HI), NUE, nitrogen response (NR), nitrogen uptake efficiency (NUP), number of filled grains per panicle (FGP), number of spikelets per panicle (SP), panicle length (PLG), plant height (PHT), relative biomass (RBM), relative plant weight (RPW), relative shoot dry weight (RSW), root length (RL), spikelet fertility percentage (SFP), spikelet per primary panicle (SPY), and thousand-grain weight (TGW). These QTLs were found across 10 chromosomes (chromosomes 1, 3, 4, 5–9, 11, and 12) except chromosomes 2 and 10. Out of the 1,064 NUE-related genes identified in this study, 327 unique genes were co-localized to these major NUE-QTL regions (Supplementary Table S17). A maximum of 110 NUE-related genes were co-localized on chromosome 3, followed by chromosome 1 and a minimum of 5 NUE-related genes each were co-localized on chromosomes 12 and 8 (Supplementary Table S17).

### Functional Evaluation Identifies a Subset of 114 NUE-Genes

To gain insights into the functional roles of 327 candidate genes identified by co-localization, we searched the OGRO database (Yamamoto et al., 2012) and found that 137 out of 327 NUE-related genes were functionally characterized (Supplementary Table 18). Of these, 70 genes were found to be associated with more than one trait. They were grouped into three categories, namely, morphological traits (148 genes), physiological traits (70 genes), and resistance or tolerance (73 genes). Maximum number of functionally characterized genes (47) were located on chromosome 1 followed by chromosomes 3, 9, 5, and the least functionally characterized gene was on chromosome 12. The chromosomes 4, 6, 7, 8, 11, and 12 had <10 candidate genes. Among the chromosomes that carried more than or equal to 10 candidate genes each totaling 114 NUE-related genes (Supplementary Table S19), we found that chromosome 1 harbors a novel hotspot with 47 genes, apart from chromosomes 3, 5, 9, and 11, which are known hotspots for NUE (Jewel et al., 2019). Overall, we found that these 4 chromosomes contribute 114 NUE-related genes, of which only 6 are validated so far, thus expanding the repertoire for the validation of their potential and further shortlisting as targets for improving NUE (Supplementary Table S19).

Interestingly, 36 of the 114 NUE-candidate genes identified here are associated with the dwarf phenotype, as their mutants are well-characterized (Supplementary Table S19). In addition, 29 genes have been characterized for the culm/leaf, 33 genes associated with the panicle/flower, 2 genes with germination, 30 with the seed/shoot seedling, and 10 with roots. Further, 17 genes had source activities such as photosynthetic activity, chlorophyll biosynthesis, starch biosynthesis, biomass, grain production, and leaf senescence, and 31 genes for biotic and abiotic stress (Supplementary Table S19).

### Shortlisting 62 NUE-Candidate Genes by Protein Interactions

To further identify the important candidates among the 114 shortlisted NUE-related genes explained above, we developed their PPI networks using Cytoscape 3.8. Only the combined score value ≥0.4 were considered for network construction. The network consisted of 80 nodes and 103 edges (Supplementary Table S20). One major cluster had 62 interacting genes (Supplementary Figure 3). We performed GO (Expath tool) as well as intensive literature search to find the biological processes and functions of these 62 genes (Supplementary Tables S21, S22). GO revealed important biological pathways like flower development, phytochromobilin biosynthetic process, protein-tetrapyrrole linkage, glutamate biosynthesis, leaf development, cytokinin metabolic process, oxidation-reduction process, auxin response, phosphorylation, response to light stimulus, pollen development and response to water deprivation (Supplementary Table S21).

**Figure 7:**
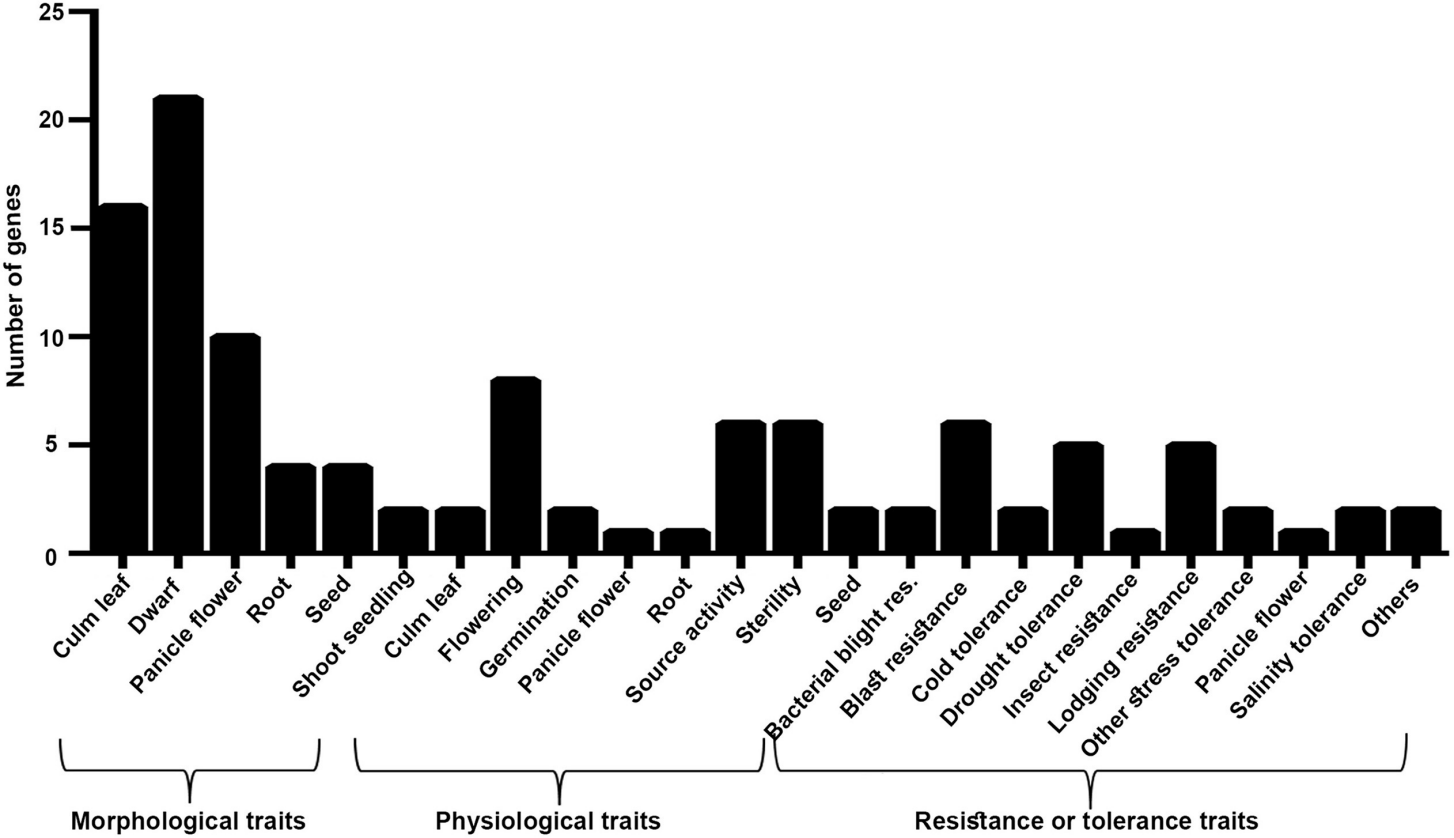
Morphological, physiological, and resistance- or tolerance-related traits associated with 62 NUE-candidate genes.

**Figure 8:**
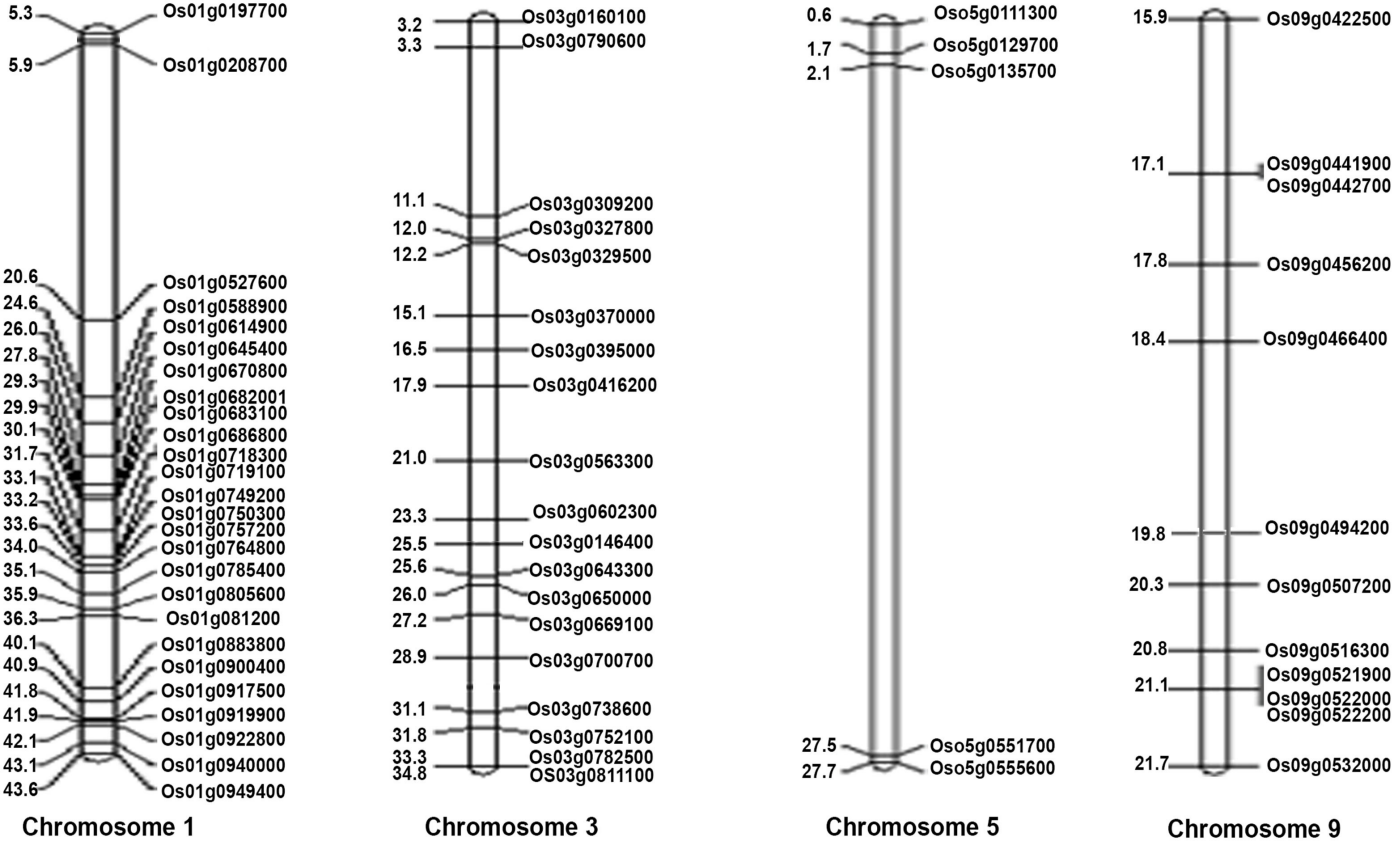
Physical positions of the 62 NUE-candidate genes on four rice chromosomes (chromosomes 1, 3, 5, and 9). Gene ID is given on the right side of the map, and the physical location of genes is given on the left side of the map (in mb).

These 62 interacting proteins include 21 genes reported for dwarf phenotype, 9 for flowering, 18 for the culm leaf (stomatal opening and transpiration cooling, chloroplast development, and stomatal density), 5 for the root, 2 for germination, 8 for source activity (including leaf senescence and chlorophyll biosynthesis), 6 for the seed, 2 for the shoot seedling, and 17 for biotic and biotic stresses (Supplementary Table S22 and Figure 7). While all these 62 genes are a subset of the 1,064 NUE-related genes, most of them have not been directly validated for NUE, except *DEP1*. Two of them (*OsRRMH* and *Gn1a*) interact with *DEP1*, a hitherto unknown observation in relation to NUE. Interestingly, the network consists of many hubs that have a larger number of interactions than *DEP1*, such as *OsATG7* with the highest number of interactions (8), followed by *SGR* (7) *OsPAO, LOX2, OsNAP* (6), *CHL9, cwa1, GH3.1, Se13, PHYB* (5), *SHL2, CHL1, OsLOX1, NADH-GOGAT1* (4), and *bc15, OAT, OsIPT7, RGB1* (3). Such hubs could play a far more important role in regulating the NUE phenotype than those genes that are least connected in the network. Chromosomal localization of these 62 NUE-candidate genes revealed the maximum number of 26 NUE-candidate genes on chromosome 1, 19 NUE-candidate genes on chromosome 3, 12 NUE-candidate genes on chromosome 9, and 5 NUE-candidate genes on chromosome 5 (Figure 8).

### *In silico* Expression Analysis of Shortlisted Candidate Genes

The expression profiles of 62 candidate genes shortlisted for NUE were checked by using microarray data from different tissues and stages of the rice life cycle using the Rice Expression Profile Database (RiceXpro). Of these, only 60 candidate genes were differentially expressed in both vegetative and reproductive tissues. *OsMADS8* was highly upregulated in anther, pistil, and endosperm, while *OsCesA9* was highly upregulated in the stem at the ripening stage and in lemma and palea. Further, *RGB1* was highly upregulated in the root. *BC6* was highly upregulated in the stem at ripening stage and in lemma and palea. The gene *phdk* was highly upregulated in the stem at the ripening stage and in lemma and palea. *OsSSl2* was highly upregulated in the leaf blade at vegetative, ripening and reproductive stages. *OsRZFP34* was highly upregulated in the stem at the ripening stage, lemma, and palea. *OsbZIP72* was highly downregulated in the leaf blade at vegetative, ripening, and reproductive stages, while *OsDWARF1* was highly downregulated in the root at vegetative and reproductive stages and also in anther and endosperm. *GH3-2* was highly downregulated in the leaf blade at vegetative and ripening stages, in the stem at the ripening stage, and in the ovary and endosperm. *GOGAT2* was upregulated in the leaf blade but highly downregulated in the endosperm and ovary. *DEP1* was moderately upregulated in the panicle and the stem but moderately downregulated in the leaf blade and ovary. This gene is well-known for NUE. The remaining 51 genes were under low or moderate regulation (Supplementary Figure 4). These expression patterns provide a molecular basis for understanding the physiology of N-responsive growth and yield toward NUE.

### Feature Selection Converges With Hierarchical Approach and Aids in Further Shortlisting

As an independent approach for shortlisting NUE-related genes, the Feature Selection Analysis with the recursive feature elimination method was used to rank the 1,064 NUE-related genes based on their microarray expression data for N-response (log_2_FC). This approach eliminated 686 features/gene IDs as non-significant features out of 1,064 and identified 378 features/IDs as significant, of which 237 genes were upregulated and 141 were downregulated in response to N (Supplementary Table S23). Among molecular targets, 92 TFs, 38 kinases, 22 transporters, 15 miRNA targets, and 2 phosphatases were found common for these 378 genes shortlisted by Feature Selection. Interestingly, 112 of them were common with the 327 NUE-genes by QTL co-localization, while 44 were common with the shortlisted 114 NUE-genes. Further, when 62 of the 114 NUE-genes that figured in the PPI networks were compared, 21 of them were common with those identified by Feature Selection (Supplementary Table S23). Genes that share commonality with QTL-co-localized genes, their shortlisted interacting partners, and molecular targets (especially, TFs) could be of great interest. For this purpose, common genes among them were searched and only six genes were found to be common to the 62 shortlisted NUE-candidates by a hierarchical method (Supplementary Table S23 and Figure 6). They are *OsZHD1* (Os09g0466400), *OsDDM1a* (Os09g0442700), and *MADS24* (Os09g0507200) on chromosome 9, and *OsARF2* (Os01g0670800) and *GAMYB* (Os01g0812000) on chromosome 1, and *OSKN2* (Os05g0129700) on chromosome 5. These six genes may be considered as high priority targets among the shortlisted gene candidates to be validated for NUE. Thus, Feature Selection Analysis independently identified one-third of the common genes at every stage of hierarchical shortlisting and offered six high-priority target genes for NUE.

### Contrasting Genotypes Differ in Photosynthesis and Transpirational Parameters

**Figure 9:**
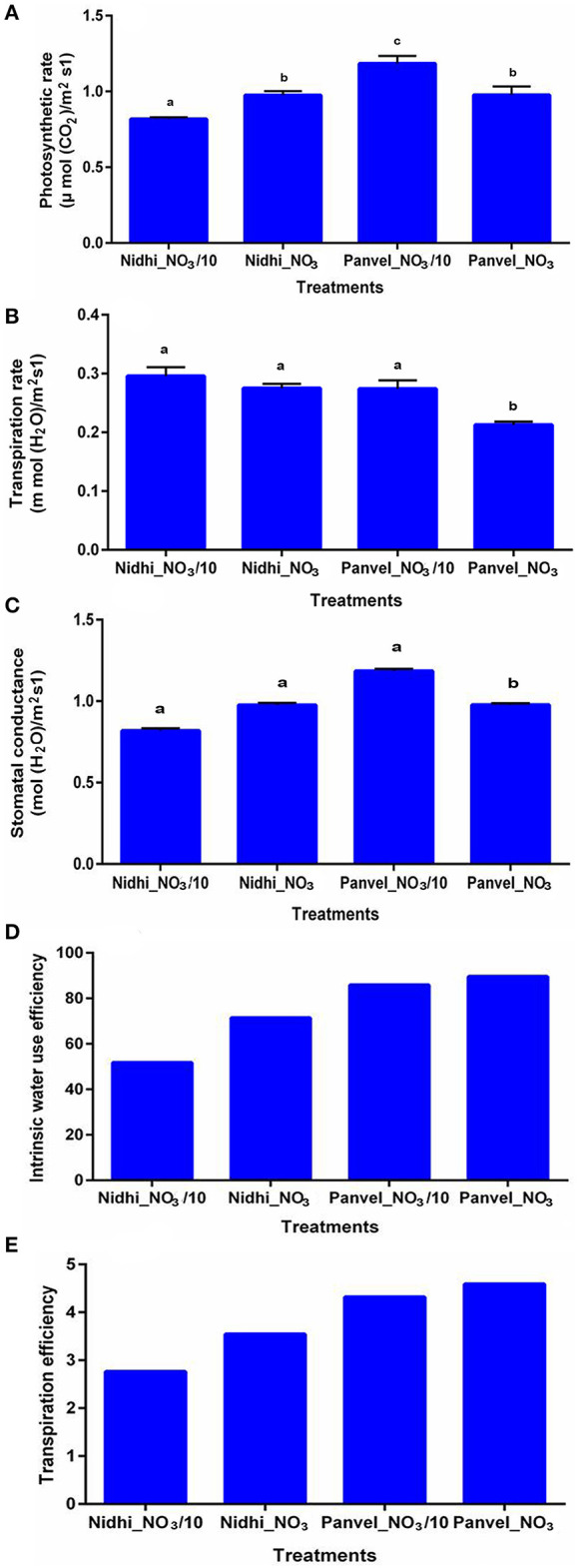
Validation of biological processes: validation was done by using Licor 6400XT (LI-COR, Lincoln, NE, USA) on 21-day-old grown plants. Plants were grown in garden soil and fertilized with Arnon–Hoagland medium having nitrate as the sole source of N with 15 mM N concentration as control while 1.5 mM was used as a test. Measurement was done in five biological **(A)** Photosynthesis was measured in terms of μ mol CO_2_/m^2^s^1^, **(B)** Transpiration was measured in terms of mol (H_2_O)/m^2^s^1^, **(C)** Stomatal conductance was measured in terms of mol(H_2_O) m^−2^ sec^−1^, **(D)** Internal water-use efficiency was measured in terms of μ mol CO_2_/mol(H_2_O), and **(E)** Transpiration efficiency was measured in terms of μ mol CO_2_/m mol H_2_O/m^2^/s. The different alphabets over bars show their statistical significance.

To experimentally validate some of the physiological processes associated with the 1,064 NUE-related genes as well as the 62 shortlisted candidate genes, 2 contrasting rice genotypes were chosen based on their NUE (Supplementary Tables S3, 21, 22; Sharma et al., 2018, 2021). Photosynthesis was found to be significantly higher for the higher NUE genotype Panvel1, relative to the lower NUE genotype Nidhi (*p* < 0.05). Interestingly, photosynthesis was significantly higher at a lower nitrate dose than the normal dose in Panvel1, whereas it was the opposite in the case of Nidhi (*p* < 0.05) (Figure 9A). Transpiration and stomatal conductance were found to be significantly higher for Nidhi than Panvel1 at the normal dose of nitrate, though not significant at lower N dose (*p* < 0.05, Figures 9B,C). Interestingly, intrinsic water-use efficiency and transpiration efficiency were higher for Panvel1 than Nidhi and were also slightly higher at the normal dose of nitrate than the lower N dose for both genotypes (Figures 9D,E).

## Discussion

Nitrogen-use efficiency is agronomically best measured as yield per unit N input, which is a derivative of two biological functions, N-response and yield. A large number of genes for N-response and/or yield have been reported in the literature, but their interface for NUE remains underexplored. Therefore, this meta-analysis was undertaken to identify the genes at the intersection of N-response and yield and to use them to shortlist the candidate genes for NUE as shown in the flowchart (Figure 1). To the best of our knowledge, this is the first comprehensive meta-analysis for NUE in any crop, except for a limited analysis of genes in the NUE-QTL regions done in barley (Han et al., 2016). Out of 16 whole transcriptome microarray data sets available for N-response in rice, seven are for nitrogen (GSE4409; Beatty et al., 2009; Perrin et al., 2013; Coneva et al., 2014; GSE59438; Hsieh et al., 2018; Takehisa and Sato, 2019), one for nitrate and urea (GSE140257), one for ammonium nitrate (Beier et al., 2018), two for nitrate (Jangam et al., 2016; Pathak et al., 2020), one for thiourea (GSE71492) and four related to macronutrients NPK (Takehisa et al., 2015; Takehisa and Sato, 2019). Together, they revealed 14,791 N-responsive genes, with several hundreds of common DEGs and thousands of unique DEGs. While the latter may reflect the differences in tissues and N-treatments, their interface with yield was of main interest for NUE in agronomic terms, especially considering that different N-forms may coexist under field conditions. Therefore, all the 14,791 N-responsive genes were used for Venn selections with 1,842 yield-related genes compiled for this study.

Despite the findings of the differences in the number of yield-related genes among the different N-responsive transcriptomic data sets generated under different growth and treatment conditions, taking them together, we identified 1,064 genes as both N-responsive and yield-related. Meta-analysis allows such identification from diverse data sets as reported earlier for Arabidopsis (Zhang et al., 2020a; Yang et al., 2021) and rice (de Abreu-Neto and Frei, 2016; Muthuramalingam et al., 2017; Cohen and Leach, 2019; Kong et al., 2019). In order to minimize variations in data processing, we used uniform selection criteria (Log_2_FC ≥ 1, value of *p* ≤ 0.05) with a default redundancy removal setting for all the microarray data sets. We designated the 1,064 genes that were both N-responsive and yield-related as NUE-related genes, analyzed, and shortlisted them using multiple bioinformatic approaches including clustering, classification, QTL co-localization, *in silico* network, and Feature Selection Analyses.

We also identified several novel NUE-related TFs and their binding sites, transporters, kinases, phosphatases, and miRNAs; shortlisted some candidate genes for NUE; and validated some of the underlying physiological processes in contrasting genotypes for NUE.

### NUE-Related Genes Involved in Important Biological Process or Functions

Gene ontology and MapMan analyses of 1,064 NUE-related genes revealed that the regulation of transport, transcription, phosphorylation and oxidation-reduction, photosynthesis, and transpiration were prominent biological classes of NUE-related genes (Supplementary Table S3 and Figure 3A). Out of 237 TFs encoded by NUE-related genes identified here (Supplementary Table S8), only two are known for NUE in rice, *OsDOF1* (Uedga et al., 2020) and *OsGRF4* (Sun et al., 2016). Two other TFs were found to be both N-responsive and yield-related but were not reported for NUE. These are *OsMADS57* (Guo et al., 2013) and *OsSPL14* (Srikanth et al., 2016). Thus, this study identified a comprehensive list of 237 NUE-related TFs, including 235 novel NUE candidates in rice, for further validation and shortlisting (Supplementary Table S8). Among the 50 enriched binding sequences/motifs identified in the upstream sequences of 1,064 NUE-related genes, only few motifs were reported to be involved in functions other than N-response and yield (Supplementary Table S9). They include Fe deficiency responses (Kobayashi et al., 2014), defense-response (Han et al., 2015), and GA biosynthesis or cold acclimation response (Jung et al., 2010). These 50 candidate motifs need the validation of their role in NUE for crop improvement.

OsbHLH TFs are known for their roles in regulating grain length, internode elongation, plant height, and yield in rice (Heang and Sassa, 2012a,b,c; Yang et al., 2018; Lee et al., 2020; Seo et al., 2020). In the present study, we proposed a model for the functional role of OsbHLH079 and its target genes in carbon–nitrogen metabolism (Figure 4B). Functional annotation of the 538 target genes of OsbHLH079 revealed that they mainly belong to nitrogen and carbon metabolism. *AMT1.1*, which transports ammonium and thus acts as the source of nutrient supply, has already been reported in N-response and yield (Ranathunge et al., 2014). Further, 47 of the 538 target genes were involved in yield, a sink activity, which suggests an important role of this TF in regulating the source–sink relationship for yield and NUE. This interpretation is consistent with the finding that OsbHLH079 regulates leaf angle and grain length (Seo et al., 2020). Therefore, this TF could be an important candidate to improve NUE, as its target genes regulate source to sink balance.

Nitrogen is mainly taken up by the plant in the form of either nitrate or ammonium ions, amino acids, or urea through their respective families of transporters. In the present study, out of 85 transporters encoded by NUE-related genes (Supplementary Table S14), only 5 are known to be related to NUE. They are *OsNPF6.1* (Tang et al., 2019), *OsNRT2.3* (Fan et al., 2016), *OsNRT2.1* (Chen et al., 2016), *OsPTR9* (Fang et al., 2013), and *OsNRT1.1A* (Wang et al., 2018). Three other transporters were separately known to be related to N response and yield but not reported for NUE. They are *OsNPF7.2, AMT1.1*, and *NPF7.4* (Ranathunge et al., 2014; Wang et al., 2018; Huang et al., 2019). Thus, this study provides a comprehensive list of NUE-related transporters in rice, including 80 novel candidates for further validation of their role in NUE (Supplementary Table S12).

Nitrogen-use efficiency can be improved by miRNA *via* the attenuation of gene expression at the post-transcriptional level or *via* translational inhibition (Zuluaga and Sonnante, 2019). Our functional annotation of the target NUE-genes for miRNAs revealed their involvement in amino acid metabolism, an important target for NUE. None of the 44 miRNAs found in the present study were previously known for NUE in rice, making them novel candidates for further validation of their role in NUE (Supplementary Table 14).

### NUE-Related Kinases and Phosphatases

Protein phosphorylation and dephosphorylation are known to be involved in N-responsive gene expression (Xiong et al., 2013), and also in post-translational modulation of N-assimilatory enzymes (Ahn et al., 2015; Waqas et al., 2018), among other functions. All the 91 kinases we reported here are novel candidates hitherto unknown for their role in NUE (Supplementary Table S18). Phosphatases are also known to be N-responsive in many crops (Kumari and Raghuram, 2020). We identified nine phosphatase-encoding genes, including fructose-1,6-bisphosphatase, which was recently reported to be potentially involved in NUE (Waqas et al., 2018). Thus, this study identified eight phosphatases as novel candidates for the validation of their role in NUE (Supplementary Table 15).

### NUE-Related Interacting Proteins

Protein networks related to N-response have been developed earlier (Fukushima et al., 2014; Obertello et al., 2015; Pathak et al., 2020). But this was the first attempt to build PPI networks for NUE-related genes to the best of our knowledge. Process annotation of most of the interactors indicated the underlying links that balance plant growth and carbon and nitrogen metabolism, which are important for NUE (Supplementary Table S16). Earlier studies showed that an unbalanced carbon–nitrogen metabolic status can result in poor plant growth, inferior photosynthetic capacity, lower nitrogen transferability, and decreased yield (Kusano et al., 2011; Bao et al., 2014). Moreover, increased carbon-nitrogen utilization rates have been linked to better photosynthesis and NUE in rice (Ju et al., 2021). Two other interactors, *NPH1* and *LAC13/14*, were not a part of the 1,064 NUE-related genes identified for this study, even though their functional annotation indicated their role in photosynthesis, flowering, and stress. They may have their post-translational role in regulating NUE.

### Hierarchically Shortlisted Candidate Genes for NUE

Nitrogen-use efficiency is a quantitative trait controlled by multiple genes and the co-localization of NUE genes to the NUE-QTLs provides important candidate genes for crop improvement. Unfortunately, previous attempts in rice followed different approaches with varying results, focusing either on N-response or on yield but not both together, as was done in this study. Chandran et al. (2016) reported the co-localization of N-responsive genes to yield-related QTLs, rather than to NUE-related QTLs in rice. Sinha et al. (2018) targeted NUE-QTLs for co-localization, but they have used only N-responsive genes independent of their role in yield. Others who focused on characterizing the NUE-QTLs reported that all the genes co-localized in those regions regardless of their actual involvement in either N-response or yield or both (Jewel et al., 2019; Mahender et al., 2019). Our approach in this study was more robust, not only because we used only those genes that are both N-responsive and yield-related but also because we targeted only the major QTLs for NUE to shortlist target genes. Based on this, we found that chromosome 1 is a hotspot with 26 NUE-related genes (Figure 6), of which only 2 were reported earlier (Chandran et al., 2016). The next hotspot we found was chromosome 3, which was earlier reported to have the most QTLs for NUE in rice by Jewel et al. (2019), but they did not report chromosome 1.

Interestingly, chromosomes 1 and 3 continued to be hot spots for NUE even after our hierarchical shortlisting of 1,064 NUE-related genes to 62, based on QTL co-localization, their functional validation in the literature, and choosing chromosomes with more than 10 of these genes followed by PPI network analysis (Figure 6). Chromosomes 5 and 9 were the next most important hotspots for NUE-related genes, as reported by Jewel et al. (2019). The importance of our shortlisting is that it resolved the similarities and differences in the genes/loci/chromosomes reported as important for N-response/NUE by different authors (cited above and detailed in Supplementary Table S17), by using additional functional parameters. Another evidence of the robustness of our shortlisting approach is that it retained some of the well-characterized genes involved in NUE till the very end, such as DEP1 (Sun et al., 2014). More importantly, we added 60 other NUE-candidate genes, 26 of which are located only on chromosomes 1 and 19 on chromosome 3, making them extremely valuable targets for breeders. Further, protein interaction networks of 62 candidate genes revealed that some of them are major functional hubs and may be more crucial for NUE in comparison to others. Chromosome 1 seems particularly attractive with 26 NUE-candidate genes, but since they span 81% of the whole chromosome, further narrowing down of the hotspot for NUE would be needed before it can be used by breeders.

### Traits Associated With NUE-Candidate Genes

Among 62 NUE candidates, 17 genes regulate more than one trait (morphological, physiological, biotic, or abiotic), while multiple genes regulate the same traits, as expected for polygenic traits like NUE. In view of the just emerging details of the NUE phenotype (Sharma et al., 2021) relative to the well-characterized nature of yield-related traits, there was scope for the identification of NUE-associated traits through yield for these 62 NUE-candidate genes. Among the morphological traits, we found that 21 genes regulate dwarf phenotype, including the well-known NUE gene, *DEP1* (Sun et al., 2014), in rice. In addition, we found 16 NUE-candidate genes for the culm leaf, 10 for the panicle flower, 4 for the root, 4 for the seed, and 2 for the shoot seedling (Supplementary Table S22 and Figure 7). Among the physiological traits, we found eight NUE-candidate genes for flowering, six for source activity, two each for the culm leaf and the seed, 1 each for the panicle flower and the root (Supplementary Table S22 and Figure 7). Among them, *Osppc4* has been reported for ammonium assimilation in leaves (Masumoto et al., 2010). Similarly, we found 29 genes associated with traits for stress, including *NADH-GOGAT1* (Tamura et al., 2010).

Thus, barring *DEP1, NADH-GOGAT1*, and *Osppc4*, the rest of the gene-trait combinations are novel for NUE. They include some traits reported for the NUE in rice, for which the genes were not known, such as panicle number, tiller number, root-related trait (Selvaraj et al., 2017), senescence (Masclaux-Daubresse et al., 2010), flowering (Devika et al., 2018), and germination (Sharma et al., 2018). They are also in line with our recent shortlisting of six vegetative traits for NUE in rice, namely, germination and flowering time, plant height, shoot length, root length, and biomass (Sharma et al., 2021). In addition, we reported here five genes, namely, *PhyB, Osbzip72, OsDREB1A, OsDREB1B*, and *OsOAT*, to be related to NUE, which were earlier reported to be important in drought tolerance (Ito et al., 2006; Lu et al., 2009; Liu et al., 2012; You et al., 2012). This indicates the crosstalk between abiotic stress response and NUE, an underexplored area as reviewed in a previous study (Jangam et al., 2016). A focused approach in this area in the future will help in crop improvement for both drought tolerance and NUE.

### NUE-Candidate Genes by Feature Selection Analysis

Feature Selection is a well-known computational ranking method to identify the best among the features in any large data set (Abeel et al., 2009). It has never been used for shortlisting gene candidates for NUE, though we used it recently to rank and shortlist phenotypic parameters for the NUE in rice (Sharma et al., 2021). In the present study, Feature Selection Analysis of the 1,064 NUE-related genes independently identified many genes, about one-third of which were common to those identified by hierarchical shortlisting at every stage (Figure 6). This convergence between the two approaches may be of particular interest for future validation. Six of the common genes are a subset of the final shortlist of 62 NUE candidates identified by a hierarchical method and can be considered high priority targets among the shortlisted genes to be validated for NUE (Supplementary Table 23).

### Photosynthesis and Transpiration Among Processes Related to NUE

Photosynthesis and transpiration were among the important physiological processes for NUE, both among the initial list of 1,064 NUE-related genes as well as in the 62 hierarchically shortlisted NUE-candidate genes. Higher NUE was reported with increased water-use efficiency in rice (Xue et al., 2013). Similarly, an increased rate of photosynthesis was associated with increased water-use efficiency in tobacco (López-Calcagno et al., 2020). All three processes, that is, photosynthesis, water-use efficiency, and NUE, were not known to operate together for NUE. Using two rice genotypes with contrasting NUE based on our earlier studies (Sharma et al., 2018, 2021), we confirmed here that high NUE variety of rice (Panvel1) shows better photosynthetic performance, transpiration efficiency, and internal water-use efficiency, as compared to low NUE variety, Nidhi (Figures 9A,B,D). Thus, our findings link all three processes together in rice in a hitherto unknown manner. They also suggest that the NUE-candidate genes belonging to these processes may be of particular attraction for crop improvement toward NUE. However, a detailed validation of other NUE-related genes/processes and their relative contribution to NUE will reveal the most appropriate targets for NUE improvement.

## Conclusions

We identified 1,064 rice genes common to N-response and yield from the literature and analyzed them in functional terms as well as for their localization on NUE-QTLs to identify chromosomal hotspots, to identify underlying processes, and to shortlist candidate genes for NUE in rice. They include many TFs, transporters, phosphatases, kinases, miRNAs, and some of the physiological processes associated with photosynthetic and transpirational processes apart from other physiological processes. Hierarchical shortlisting approach yielded 62 candidate genes for NUE, one-third of which were also identified by the Feature Selection approach, apart from offering a subset of six genes as high-priority targets for the validation of their role in crop improvement for NUE.

## Data Availability Statement

The original contributions presented in the study are included in the article/Supplementary Material, and other data sources used for this meta analysis have been duly cited; further inquiries can be directed to the corresponding author/s.

## Author Contributions

SK and NS compiled yield-related genes, constructed networks, and analyzed TFs. SK analyzed functional annotations, subcellular, chromosomal, and QTL co-localization of genes, kinases, phosphatases, and their *in silico* expression, performed the functional evaluation and hierarchical shortlisting of NUE-candidate genes, and wrote the first draft of the manuscript. NS compiled and analyzed N-responsive genes from transcriptome data sets, miRNAs, transporters, and source–sink relationship for OsBHLH079 and carried out Feature Selection and experimental validation of physiological processes for NUE. NR conceived the idea and guided the work, helped in data interpretation, and edited and finalized the manuscript. All authors contributed to the article and approved the submitted version.

## Conflict of Interest

The authors declare that the research was conducted in the absence of any commercial or financial relationships that could be construed as a potential conflict of interest.

## Supplementary Material

Supplementary Figure 1 **(A)** Metabolism overview of nitrogen-use efficiency- (NUE-)related genes in MapMan. **(B)** Cell response overview of NUE-related genes in MapMan. Red and blue color boxes represent the upregulated and the downregulated genes, respectively. **(C)** Subcellular localization of 1,064 NUE gene products.

Supplementary Figure 2 Protein interaction network analysis of 1,064 NUE-related genes. **(A–R)** shows sub clusters identified by using plugin MCODE. Sub-clusters have been arranged according to MCODE score.

Supplementary Figure 3 Protein interaction network of NUE-candidate genes. One main transcriptional regulatory network having 62 interacting NUE-genes including dense and erect panicle (DEP1). Red and green nodes represent the upregulated and downregulated differentially expressed genes (DEGs), respectively.

Supplementary Figure 4 *In silico* expression analysis of 62 NUE-candidate genes using Rice Expression Profile Database (RiceXPRO).

Supplementary Table S1 Details of the 16 rice N-responsive microarray transcriptomic data sets used in this study.

Supplementary Table S2 NUE-related genes (1,064) along with their chromosomal locations, Log_2_FC value (from N-responsive transcriptome data), *p*-value, traits, keywords, and references.

Supplementary Table S3 Gene ontology- (GO-) based functional annotation of 1,064 NUE-related differentially expressed genes (DEGs; value of *p* ≤ 0.05) by Expath 2.0.

Supplementary Table S4 MapMan-based regulation overview of 1,064 NUE-related DEGs.

Supplementary Table S5 MapMan-based metabolism overview of 1,064 NUE-related DEGs.

Supplementary Table S6 MapMan-based metabolism overview of 1,064 NUE-related DEGs.

Supplementary Table S7 Subcellular localization of 1,064 NUE-gene products.

Supplementary Table S8 Transcription factors (TFs) encoded by 237 NUE-genes, and their families, functions, and references.

Supplementary Table S9 Enriched motifs in the promoter regions (2 kB upstream) of 1,064 NUE-related genes.

Supplementary Table S10 Conserved motifs found in the promoter regions of 1,064 NUE-genes, their TF family, and GO analysis.

Supplementary Table S11 GO analysis of 538 NUE-related genes (target genes for TF OsbHLH079).

Supplementary Table S12 Major transporters encoded by 85 NUE genes, and their families, functions, and references.

Supplementary Table S13 NUE-related 44 unique microRNAs (miRNAs) and their target genes, traits, and references.

Supplementary Table S14 Details of different classes of kinases encoded by NUE-related genes and their classification and gene names based on i-TAQ database, the database by Vij et al. (2008), and Oryzabase database.

Supplementary Table S15 NUE-related gene-encoding phosphatases.

Supplementary Table S16 Cluster-wise pathway of protein among 1,064 NUE-related genes.

Supplementary Table S17 Major NUE quantitative trait loci (QTLs) (from the literature) regulating 18 unique traits onto which 327 NUE-related genes were co-localized.

Supplementary Table S18 Functionally characterized 137 NUE-related genes by OGRO database.

Supplementary Table S19 Shortlisted 114 candidate genes for NUE and their functional characterization by OGRO database.

Supplementary Table S20 Protein involved in protein–protein interaction (PPI) network among 114 NUE-related genes.

Supplementary Table S21 GO analysis of 62 interacting genes of the main network by Expath 2.0.

Supplementary Table S22 Functional characterization of 62 interacting genes of the main network through OGRO database.

Supplementary Table S23 Common NUE-genes among Feature Selection analysis and hierarchically shortlisted NUE-genes.
