# Supplementary figures and images for: Meta-Analysis of Yield-Related and N-Responsive Genes Reveals Chromosomal Hotspots, Key Processes and Candidate Genes for Nitrogen-Use Efficiency in Rice
Source: Front Plant Sci. 2021 Jun 8;12:627955. doi: 10.3389/fpls.2021.627955 (PMC8217879; doi:10.3389/fpls.2021.627955)

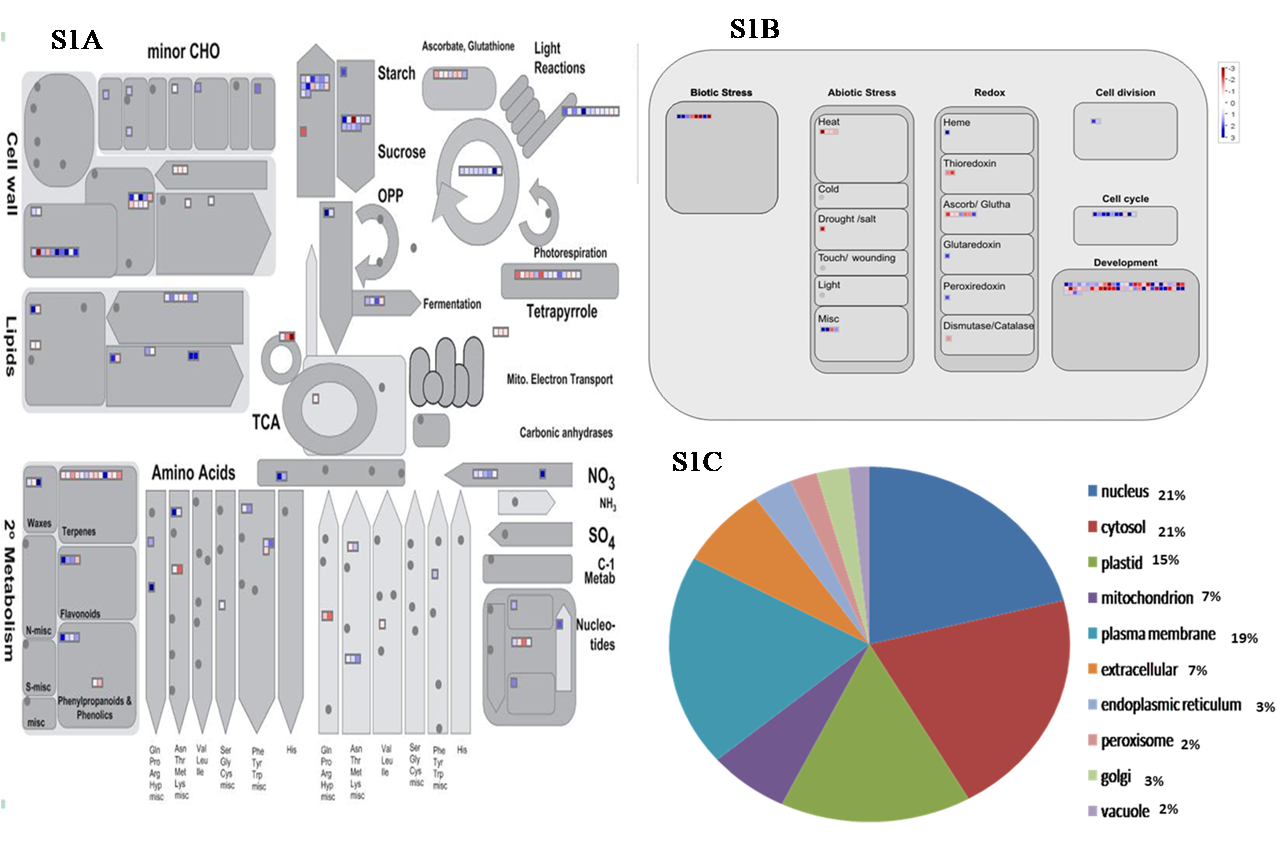

Supplement: Supplementary Figure 1 — (A) Metabolism overview of nitrogen-use efficiency- (NUE-)related genes in MapMan. (B) Cell response overview of NUE-related genes in MapMan. Red and blue color boxes represent the upregulated and the downregulated genes, respectively. (C) Subcellular localization of 1,064 NUE gene products. [file Image_1.TIF]

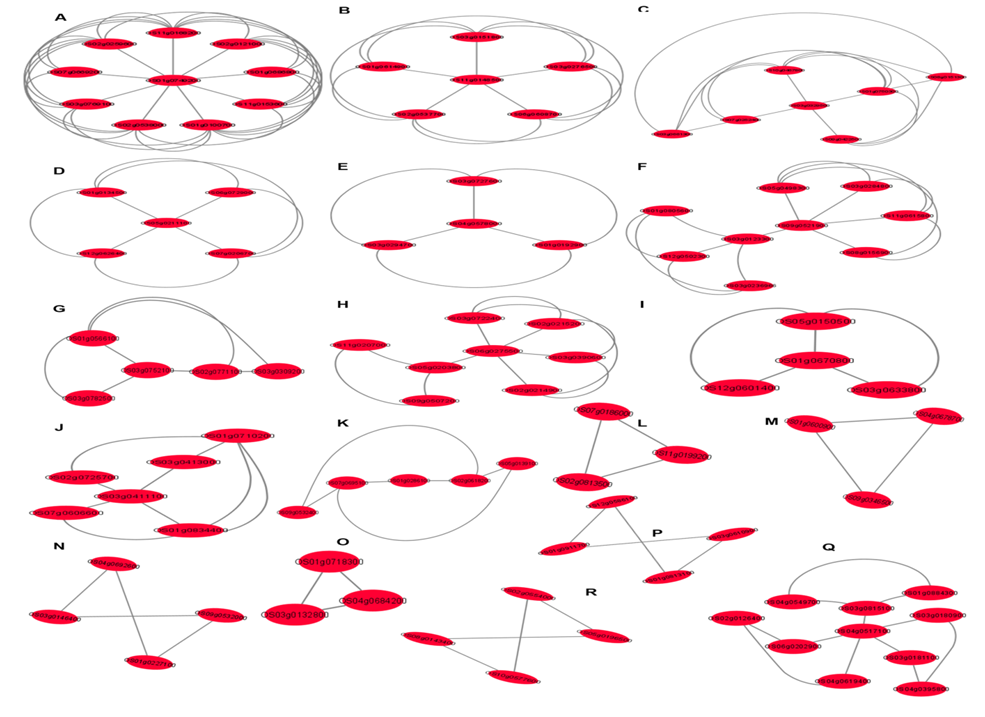

Supplement: Supplementary Figure 2 — Protein interaction network analysis of 1,064 NUE-related genes. (A–R) shows sub clusters identified by using plugin MCODE. Sub-clusters have been arranged according to MCODE score. [file Image_2.TIF]

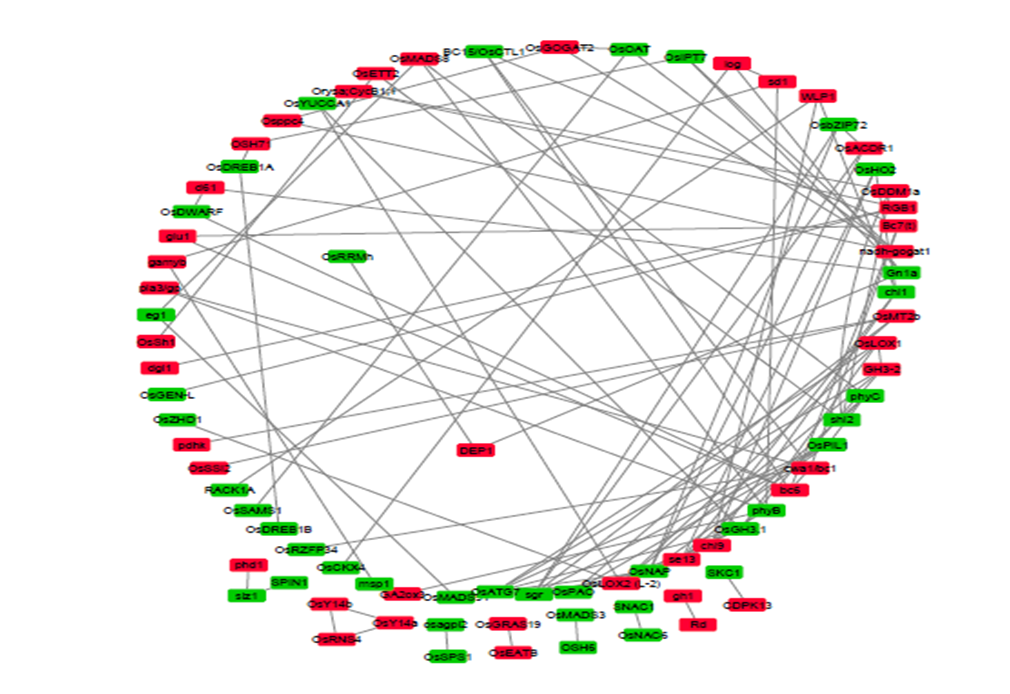

Supplement: Supplementary Figure 3 — Protein interaction network of NUE-candidate genes. One main transcriptional regulatory network having 62 interacting NUE-genes including dense and erect panicle (DEP1). Red and green nodes represent the upregulated and downregulated differentially expressed genes (DEGs), respectively. [file Image_3.TIF]

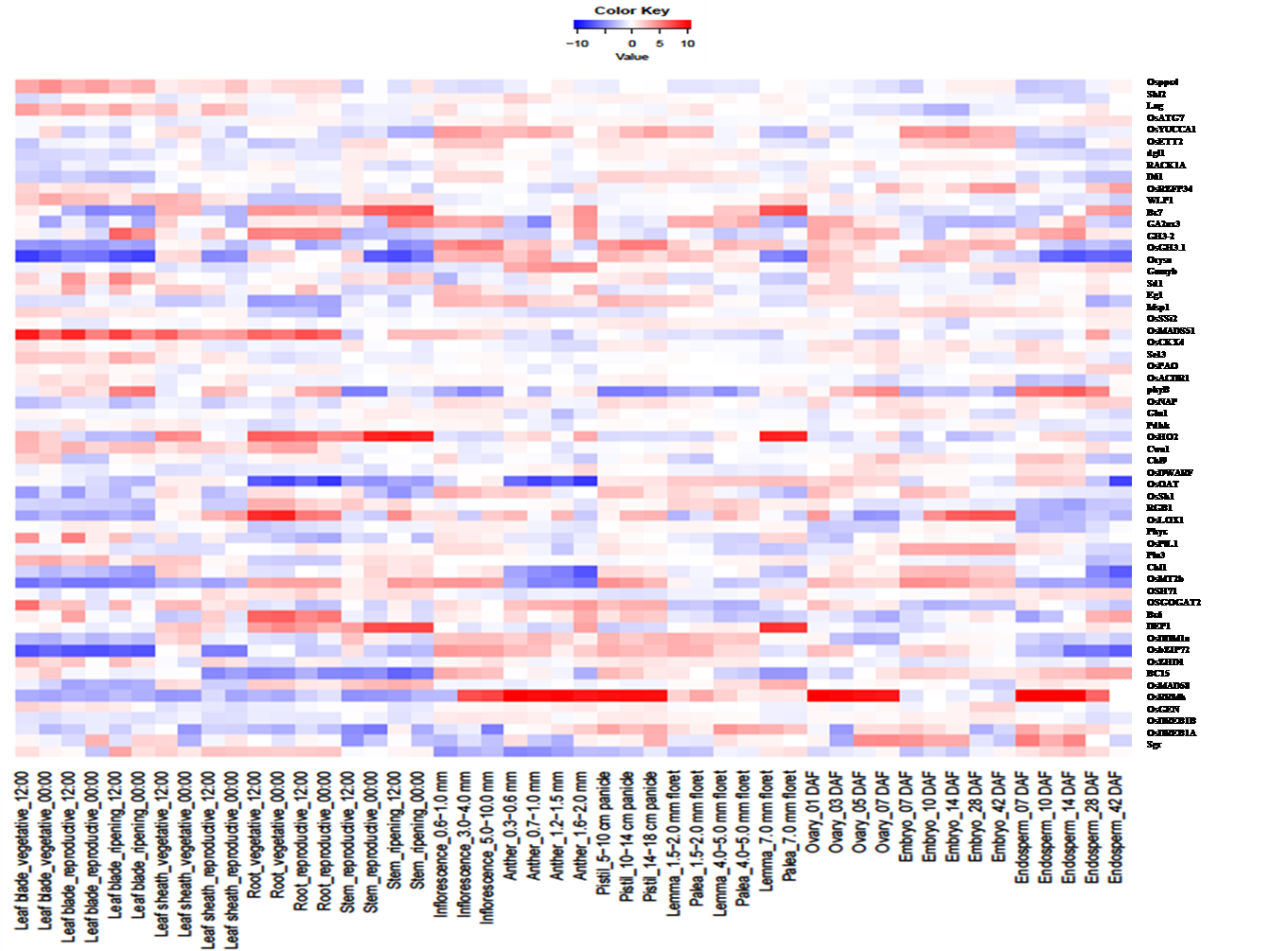

Supplement: Supplementary Figure 4 — In silico expression analysis of 62 NUE-candidate genes using Rice Expression Profile Database (RiceXPRO). [file Image_4.tif]
